# MPAC: a computational framework for inferring pathway activities from multi-omic data

**DOI:** 10.1093/bioinformatics/btaf490

**Published:** 2025-09-11

**Authors:** Peng Liu, David Page, Paul Ahlquist, Irene M Ong, Anthony Gitter

**Affiliations:** Department of Biostatistics and Medical Informatics, University of Wisconsin–Madison, Madison, WI 53726, United States; Carbone Cancer Center, University of Wisconsin–Madison, Madison, WI 53792, United States; Department of Biostatistics and Medical Informatics, University of Wisconsin–Madison, Madison, WI 53726, United States; Carbone Cancer Center, University of Wisconsin–Madison, Madison, WI 53792, United States; Department of Computer Sciences, University of Wisconsin–Madison, Madison, WI 53706, United States; John and Jeanne Rowe Center for Research in Virology, Morgridge Institute for Research, Madison, WI 53715, United States; McArdle Laboratory for Cancer Research, University of Wisconsin–Madison, Madison, WI 53705, United States; Institute for Molecular Virology, University of Wisconsin–Madison, Madison, WI 53706, United States; Department of Biostatistics and Medical Informatics, University of Wisconsin–Madison, Madison, WI 53726, United States; Carbone Cancer Center, University of Wisconsin–Madison, Madison, WI 53792, United States; Department of Obstetrics and Gynecology, University of Wisconsin–Madison, Madison, WI 53705, United States; Center for Human Genomics and Precision Medicine, University of Wisconsin–Madison, Madison, WI 53705, United States; Department of Biostatistics and Medical Informatics, University of Wisconsin–Madison, Madison, WI 53726, United States; Department of Computer Sciences, University of Wisconsin–Madison, Madison, WI 53706, United States; John and Jeanne Rowe Center for Research in Virology, Morgridge Institute for Research, Madison, WI 53715, United States

## Abstract

**Motivation:**

Fully capturing cellular state requires examining genomic, epigenomic, transcriptomic, proteomic, and other assays for a biological sample and comprehensive computational modeling to reason with the complex and sometimes conflicting measurements. Modeling these so-called multi-omic data is especially beneficial in disease analysis, where observations across omic data types may reveal unexpected patient groupings and inform clinical outcomes and treatments.

**Results:**

We present Multi-omic Pathway Analysis of Cells (MPAC), a computational framework that interprets multi-omic data through prior knowledge from biological pathways. MPAC leverages network relationships encoded in pathways through a factor graph to infer consensus activity levels for proteins and associated pathway entities from multi-omic data, runs permutation testing to eliminate spurious activity predictions, and groups biological samples by pathway activities to allow identifying and prioritizing proteins with potential clinical relevance, e.g. associated with patient prognosis. Using DNA copy number alteration and RNA-seq data from head and neck squamous cell carcinoma patients from The Cancer Genome Atlas as an example, we demonstrate that MPAC predicts a patient subgroup related to immune responses not identified by analysis with either input omic data type alone. Key proteins identified via this subgroup have pathway activities related to clinical outcome as well as immune cell composition. Our MPAC R package enables similar multi-omic analyses on new datasets.

**Availability and implementation:**

The MPAC package is available at Bioconductor https://bioconductor.org/packages/MPAC

## 1 Introduction

Cancer is a complex set of diseases with a great diversity of genomic aberrations and altered signaling pathways (Hanahan 2022). The Cancer Genome Atlas (TCGA) generated data spanning copy number alteration (CNA), DNA mutation, DNA methylation, mRNA expression, microRNA expression, and protein expression for thousands of tumor samples, and included clinical patient outcomes, leading to many insights into the cancers that were profiled (Hoadley *et al.* 2018). In addition, this extensive multi-omic data provides clues to tumor regulation, which have led to the development of many computational methods to integrate multi-omic data to obtain comprehensive views on cancer (Stein-O’Brien *et al.* 2018b; Picard *et al.* 2021; Maghsoudi *et al.* 2022).

In particular, biological pathway-based approaches have been demonstrated as a powerful way to integrate multi-omic data [reviewed in Maghsoudi *et al.* (2022)]. Altered expression or function of different genes in the same pathway can have similar impacts on overall pathway activity. Similarly, diverse alterations of expression or function of the same gene or its protein product—e.g. through DNA mutations, CNAs, or changes in epigenetic modifications, transcript expression, or protein translation, stability, or post-translational modifications—can also suppress, stimulate, or otherwise modulate a particular pathway. These properties allow modeling based on multi-omic inputs to infer pathway activity to more accurately reflect underlying biology than modeling based on a narrow, incomplete view from a single genomic data type. Accordingly, whereas a single data type rarely contains the full explanation for oncogenesis, pathway-based approaches are a particularly advantageous way to understand cancer mechanisms and their underlying relationships.

Several notable pathway-based methods have demonstrated the benefits of multi-omic data integration for cancer interpretation. For example, Multi-omics Master-Regulator Analysis (MOMA) identified 112 distinct tumor subtypes and 24 conserved master regulator blocks across 20 TCGA cohorts (Paull *et al.* 2021). OncoSig delineated tumor-specific molecular interaction signaling maps for the full repertoire of 715 proteins in the COSMIC Cancer Gene Census (Broyde *et al.* 2021). COSMOS combined signaling, metabolic, and gene regulatory networks to capture crosstalks within and between multi-omics data (Dugourd *et al.* 2021). PAthway Recognition Algorithm using Data Integration on Genomic Models (PARADIGM) integrates multi-omic data via a factor graph to infer activities of all the proteins in a pathway network (Vaske *et al.* 2010; Sedgewick *et al.* 2013). Initially, PARADIGM was successfully applied to breast cancer and glioblastoma patients using CNA and gene expression microarray data to find clinically relevant groups and associated pathways. It was further applied to reveal multiple low-frequency but high-impact mutations in glioblastoma, ovarian, and lung cancers (Ng *et al.* 2012) and was incorporated into the standard TCGA analysis pipeline (Hoadley *et al.* 2014, 2018).

Despite such successes, there are still opportunities to further improve multi-omic modeling. MOMA and OncoSig focused on direct interactions around master regulators for transcription. The indirect effects of proteins further downstream of the master regulators in biological pathways were not considered. PARADIGM’s application across many cancer types focused on grouping patients by their inferred pathway levels or enriched pathways (Berger *et al.* 2018; Hoadley *et al.* 2018). However, in-depth analysis on the molecular basis of patient grouping, careful interpretation of its inferred pathway levels, and an end-to-end computational process were lacking. PARADIGM’s inferred pathway levels are abstract quantities indicating the log-likelihood ratio of proteins being activated or repressed, but they represent neither protein abundance nor any particular post-translational modification and cannot be experimentally measured. Other existing patient stratification methods by multi-omic data either do not use biological pathway information (Duan *et al.* 2021) or rely on unrealistically small pathways (Zhao *et al.* 2021). As a result, it is hard to identify key proteins from a broad perspective with meaningful biological interpretation and clinical implication.

Here, we develop a computational framework, named Multi-omic Pathway Analysis of Cells (MPAC), to integrate multi-omic data for understanding cellular networks. It is built upon the PARADIGM method with notable improvements including providing enhanced insights to the molecular basis and clinical implications of pathway-based patient groups as well as streamlining the whole computational process. In this work, we apply MPAC to Head and Neck Squamous Cell Carcinoma (HNSCC), which accounts for ∼500 000 deaths per year worldwide (Mody *et al.* 2021), and group patients by their significantly altered pathways. Among other results, MPAC predicts a patient group that is enriched with immune response pathways, and this group cannot be predicted from the individual omic data types alone. Investigating this group identifies seven proteins that have activated pathway levels associated with better overall survival. These findings are validated by a holdout set of TCGA HNSCC samples. We demonstrate MPAC’s improvements over PARADIGM by showing that PARADIGM cannot identify such an immune response group. We also evaluate MPAC’s robustness by running it with different settings and another TCGA cancer type, cholangiocarcinoma. Lastly, we present an interactive R Shiny app that lets users explore all the results generated from this work.

## 2 Materials and methods

### 2.1 Genomic and clinical datasets

We downloaded the TCGA HNSCC genomic datasets (Cancer Genome Atlas Network 2015) from NCI Genomic Data Commons (GDC) Data Portal version 29.0 (https://portal.gdc.cancer.gov/), which was released on 31 March 2021. Gene-level copy number scores were used for CNA and log_10_(FPKM + 1) values were used for RNA-seq. Patients’ human papillomavirus (HPV) status was obtained from their biospecimen manifest files. Patients’ clinical data was downloaded from TCGA Pan-Cancer Atlas (Liu *et al.* 2018) via https://api.gdc.cancer.gov/data/1b5f413e-a8d1-4d10-92eb-7c4ae739ed81. 492 HNSCC patients that had CNA, RNA-seq, and clinical data available were stratified by HPV status and then randomly divided into exploratory sets (71 HPV+ and 322 HPV−) and validation sets (18 HPV+ and 81 HPV−). Importantly, only the exploratory set was used for all MPAC algorithm development and refinement.

MPAC’s pathway definitions were taken from the TCGA Pan-Cancer Atlas (Hoadley *et al.* 2018), which compiled interactions from NCI-PID (Schaefer *et al.* 2009), Reactome (Gillespie *et al.* 2022), and KEGG (Kanehisa *et al.* 2023) and superimposed them into a single network. The input network for MPAC included 19 477 entities, including 7321 proteins, 9349 complexes, 2092 families, 591 abstract processes, 15 miRNAs, 82 RNAs, and 27 other types of entities. It also included 45 313 interactions containing 2133 activations and 401 repressions at the transcript level, 7723 activations and 1083 repressions at the protein level, 24 870 and 9103 memberships for complexes and families, respectively. The 2085 Biological Process Gene Ontology (GO) terms (Gene Ontology Consortium *et al.* 2023) for characterizing patient or cell line pathway alteration were downloaded from the DrugCell (Kuenzi *et al.* 2020) GitHub repository (https://github.com/idekerlab/DrugCell/blob/public/data/drugcell_ont.txt). GO terms from DrugCell had more distinct genes between parental and offspring terms because DrugCell required a parent to have ≥10 genes distinct from all child terms and have ≥30 genes more than any child. The root GO term (i.e. the ancestor of all the other GO terms), “biological process,” was not used in this study because it was not a specific functional description.

### 2.2 MPAC workflow

**Figure 1.**
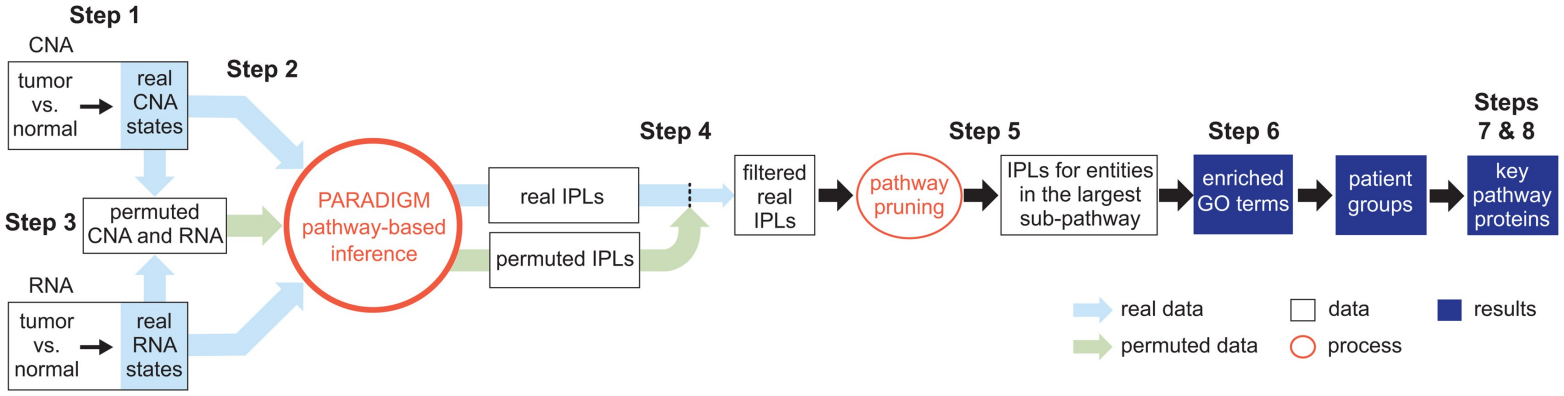
Overview of the MPAC workflow. MPAC calculates IPLs from real and permuted CNA and RNA data. It filters real IPLs using the permuted IPLs to remove spurious IPLs. Then, MPAC focuses on the largest pathway subset network with filtered IPLs to compute GO term enrichment, predict patient groups, and identify key group-specific proteins.

We developed MPAC to integrate multi-omic data to identify key pathways and proteins with biological and clinical implications, and to predict new patient groups associated with distinct pathway alterations. MPAC’s workflow contains eight steps (Fig. 1 and Note 1, available as supplementary data at *Bioinformatics* online); (Step 1) From CNA and RNA-seq data, determine genes’ CNA and RNA ternary states (i.e. repressed, normal, or activated). CNA and RNA-seq data are selected as the input multi-omic data because PARADIGM had success with them (Vaske *et al.* 2010; Sedgewick *et al.* 2013; Hoadley *et al.* 2018); (Step 2) Use CNA and RNA state together with a comprehensive biological pathway network from TCGA (Hoadley *et al.* 2018) to calculate pathway levels with PARADIGM’s factor graph model. The TCGA pathway network characterizes interactions at both transcriptional and post-transcriptional levels. PARADIGM’s inferred pathway levels are calculated not only for proteins, but also for several other types of pathway entities, such as protein complexes and gene families; (Step 3) Permute input CNA and RNA data for filtering inferred pathway level in the next step. CNA and RNA states are permuted randomly between genes in each patient. Inferred pathway levels for each pathway entity are calculated with PARADIGM from 100 sets of permuted data to build a background distribution representing inferred pathway levels observed by chance; (Step 4) Inferred pathway levels computed from real data are compared with those from permuted data to filter out inferred pathway level changes that could be observed by chance. Because both the real and permuted pathway levels are different between patients, this filtering step creates a patient-specific set of inferred pathway levels representing each patient’s unique pathway alteration profiles; (Step 5) From the remaining pathway networks, retain the connected component that contains the largest number of connected pathway entities. This focuses on the main subset of entities that are connected in the pathway network and presumably lead to similar functional alterations; (Step 6) Build patient pathway profiles and predict patient groups. Each patient’s pathway profile contains a selected set of 2085 cancer- and general biology-relevant GO terms (Kuenzi *et al.* 2020; Gene Ontology Consortium *et al.* 2023). Each GO term is characterized by an overrepresentation test *P*-value on entities selected from the previous step. *P*-values for all the GO terms are adjusted for multiple hypothesis testing and log-transformed to group patients (Lun *et al.* 2016); (Step 7) Identify key proteins that all have activated or repressed inferred pathway levels between patients from the same group; (Step 8) Compare data on key proteins with patients’ clinical data to evaluate potential impact of protein inferred pathway levels on patients’ clinical outcomes.

We provide further details about these steps here and in Note 2, available as supplementary data at *Bioinformatics* online. For TCGA data, the signs of CNA focal scores were used to define activated (positive), normal (i.e. focal score is exactly 0), or repressed (negative) CNA state as the input for MPAC. To define input RNA state, a gene’s RNA-seq expression levels from normal patient samples were fit to a Gaussian distribution. If a gene’s expression levels in tumor samples fell within two standard deviations from the mean of this distribution, the gene’s RNA state was defined as normal. Otherwise, its RNA state was repressed or activated depending on whether its expression level was below or above the two standard deviations from the mean. MPAC takes two standard deviations on a Gaussian distribution as a threshold because it corresponds to the common *P *< .05 cutoff.

MPAC ran PARADIGM in the default configuration (Vaske *et al.* 2010) except with a more stringent expectation-maximization convergence criteria of change of likelihood <10^−9^ under a maximum of 10^4^ iterations. To prepare permuted input, paired CNA and RNA states were randomly shuffled between all the genes within the patient. 100 permuted samples were prepared for each real tumor sample resulting in a total of 49 200 permuted samples for the 492 patients. This large number of computational jobs was processed through UW-Madison’s Center for High Throughput Computing (Center for High Throughput Computing 2006) with HTCondor (Thain *et al.* 2005).

A pathway entity’s inferred pathway level from a real tumor sample was set to NA if it fell within three median absolute deviations of the inferred pathway levels from the corresponding 100 permuted samples. This filtering helped to remove inferred pathway levels that could be observed by chance. Entities with non-NA inferred pathway levels were mapped to the input pathway network. The largest connected subset of the pathway network with non-NA inferred pathway levels was kept for downstream analysis. Other entities not in this largest subset had their inferred pathway levels set to zero. This allowed us to focus on the entities that act together in pathways.

After the filtering by permuted samples and the largest pathway subset, an entity’s pathway state was defined by the sign of its inferred pathway level, where a positive or negative inferred pathway level corresponded to an activated or repressed state, respectively, and a zero inferred pathway level corresponded to a normal state. Based on normal or altered pathway states, GO enrichment was calculated by Fisher’s exact test, and the *P*-values were adjusted by the Benjamini and Hochberg procedure. Similarly, GO enrichment was calculated for the CNA and RNA inputs by their normal or altered states. If other gene sets are preferred instead of GO terms, users can supply a GMT-formatted file of the gene sets to the “fgmt” option in MPAC’s “ovrGMT()” function. Patients were grouped by their adjusted *P*-values from GO enrichment using CNA, RNA, or inferred pathway levels.

## 3 Results

### 3.1 MPAC predicted an immune response HNSCC group not found by CNA or RNA-seq data alone

**Figure 2.**
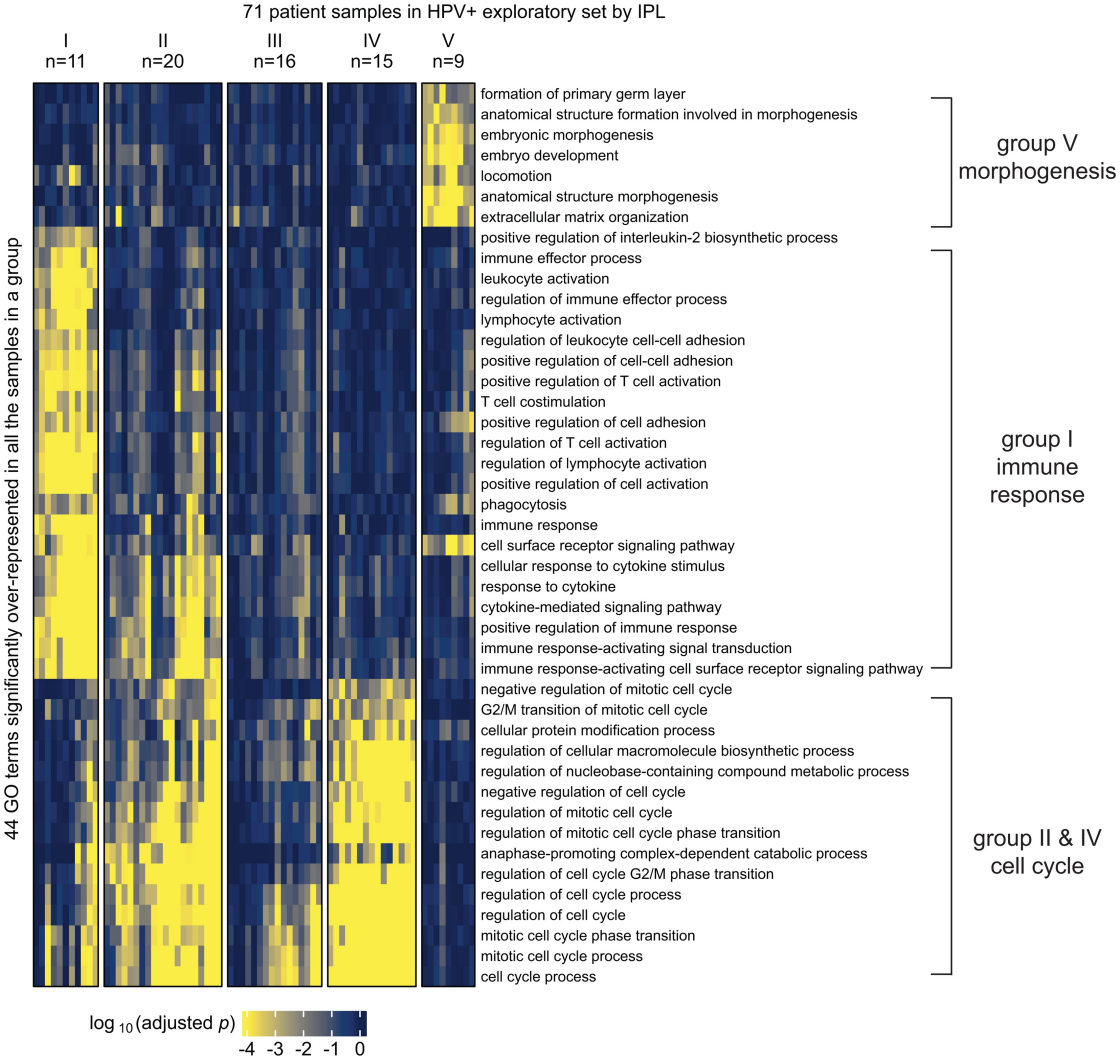
MPAC predicted functionally distinct patient groups in the HPV+ exploratory set. Patient groups were derived from GO term enrichment based on IPLs.

**Figure 3.**
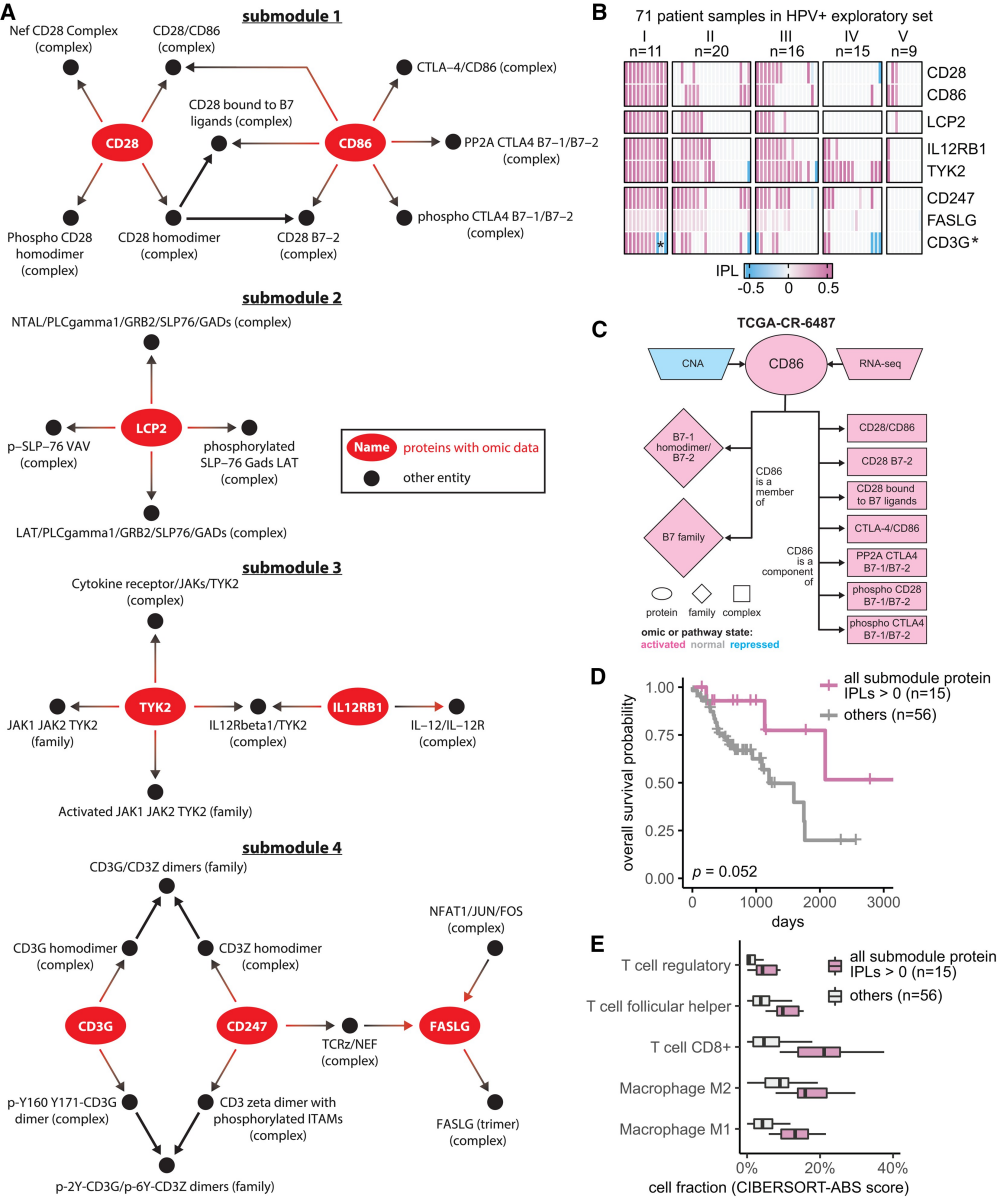
Seven proteins identified in the immune response patient group from HPV+ exploratory set associated with patient overall survival. (A) Consensus pathway submodules in the eleven immune response patient samples from group I. Proteins are colored in red and other pathway entities are black. (B) IPLs of submodule proteins in the 71 HPV+ exploratory set samples. Except for CD3G, which had both positive and negative IPLs (denoted by *) in group I, the other seven proteins had positive IPLs. (C) CNA, RNA, and pathway states of CD86 as well as pathway states of its pathway network neighbors in a group I patient sample TCGA-CR-6487. B7-1 homodimer/B7-2 (family), B7 family (family), and phospho CD28 B7-1/B7-2 (complex) do not have activated pathway state in all the 11 group I patients (Fig. 5B, available as supplementary data at *Bioinformatics* online) and thus are not included in Fig. 3a. (D) Overall survival of the HPV+ exploratory set stratified by IPLs of the seven proteins combined. (E) HPV+ exploratory set immune cell compositions stratified by IPLs of all seven proteins combined.

**Figure 4.**
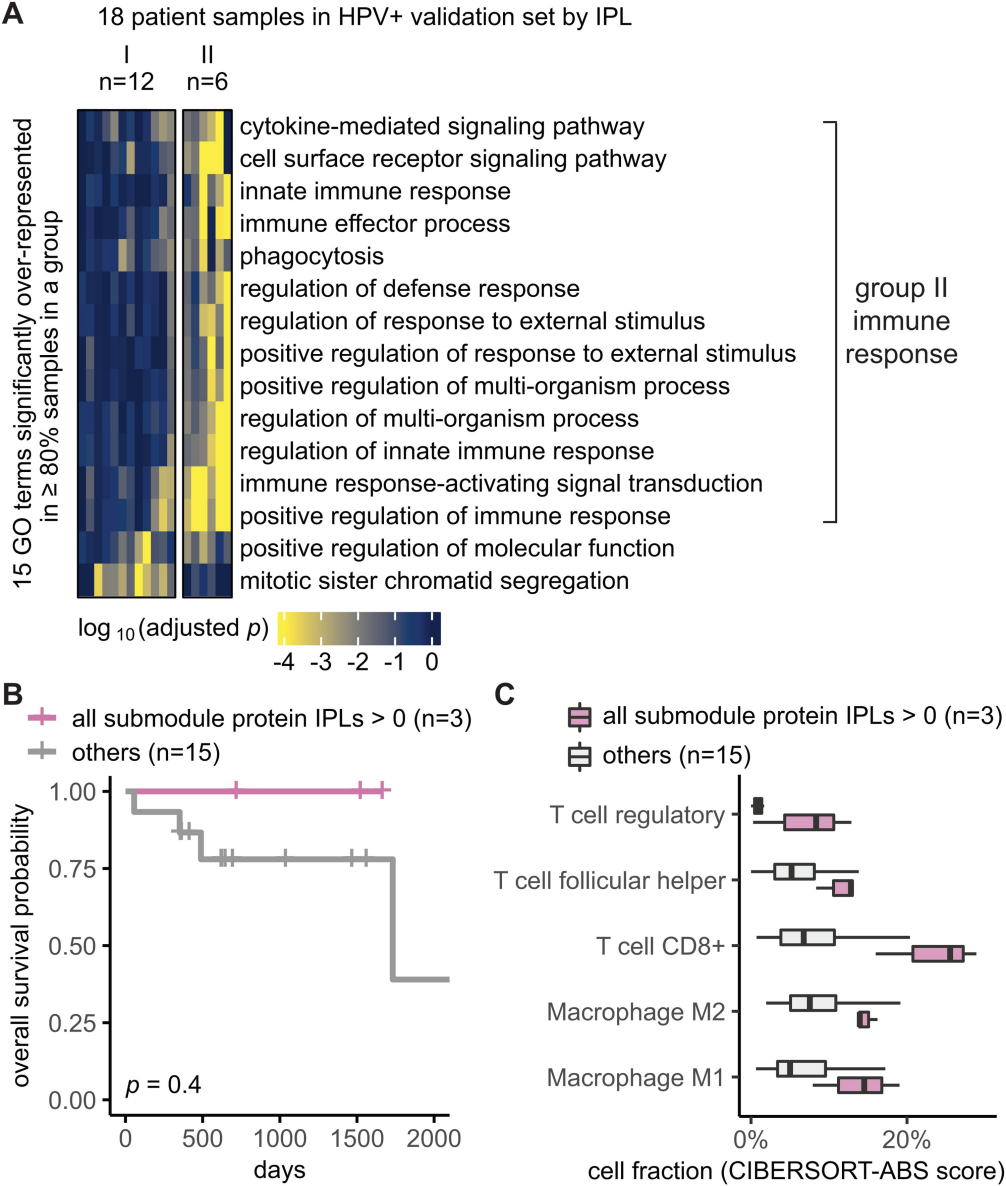
Independent validation set confirmed MPAC’s immune response group and key proteins. (A) Grouping of HPV+ validation set patient samples. The selection threshold was lowered to ≥80% to include more GO terms. (B) Overall survival of the HPV+ validation stratified by the IPLs of all the seven submodule proteins combined. (C) HPV+ validation set immune cell compositions stratified by the IPLs of all the seven proteins combined.

We applied MPAC to TCGA HNSCC patients to predict patient groups by their pathway alterations. We selected the 492 patients that had CNA, RNA-seq, and overall survival data available. Of these 492 tumors, 89 carried human papillomavirus DNA (HPV+) and 403 did not (HPV−), a distinction linked to major differences in HNSCC tumor biology and clinical treatment response (Powell *et al.* 2021). HPV+ HNSCC is mainly caused by HPV’s E6 and E7 proteins, whereas HPV- HNSCC has much higher mutation loads and distinct oncogenic pathways than HPV+. Because of such differences, we applied MPAC to the two HPV subtypes separately. We further randomly divided patients into exploratory sets (71 HPV+ and 322 HPV−) and validation sets (18 HPV+ and 81 HPV−) (Table 1, available as supplementary data at *Bioinformatics* online). Our goal was to first tune MPAC and identify pathway-based patient groups in the exploratory set and then test our discoveries in the validation set. MPAC identified five groups from each HPV subtype based on the patient pathway profiles (Fig. 2 and Fig. 1A, available as supplementary data at *Bioinformatics* online). For HPV+ patients (Fig. 2), four of the five groups had distinct pathway features. Group I patients had alterations mainly in immune response pathways, groups II and IV in cell cycle pathways, and group V in morphogenesis pathways. Group III had pathway alterations in some patients but did not show an obvious biologically meaningful consensus profile. For HPV- patients (Fig. 1A, available as supplementary data at *Bioinformatics* online), three of the five patient groups had distinct pathway alterations: groups I and IV in cell cycle pathways and group III in immune response pathways. Groups II and V did not show obvious consensus pathway features. The distinct pathway features for many of the patient groups suggested that MPAC is capable of building biomedically relevant patient pathway profiles and predicting patient groups. Further analysis showed that the same groups and pathways cannot be recovered from analysis of individual omic data types alone (Note 3 and Figs 1–4, available as supplementary data at *Bioinformatics* online).

### 3.2 Proteins from the HPV+ immune response group associated with patient overall survival

Given that MPAC discovered an immune response patient group that could not be found by CNA or RNA-seq data alone, we were interested in activated or repressed pathway submodules and key proteins shared by the eleven patients in this group. We defined a pathway submodule as a pathway subset containing ≥5 entities, at least one of which was a protein with input omic data. We required that all submodule entities must have activated or repressed inferred pathway levels in the eleven patients. Four such submodules were identified (Fig. 3A). They contained five to twelve entities and collectively eight proteins (Fig. 3A, red ovals). Seven of these proteins, CD28, CD86, TYK2, IL12RB1, LCP2, FASLG, and CD247, had activated inferred pathway levels in all eleven group I patients (Fig. 3B), suggesting a consensus functional role across patients within this immune response group. Interestingly, prior studies collectively showed that gene expression levels of the seven proteins except for LCP2 are associated with immune infiltration in HNSCC (de Vos *et al.* 2020; Chen *et al.* 2022; Chi *et al.* 2022; He *et al.* 2022; Wang *et al.* 2022; Zhu *et al.* 2022). Below we show a similar association for the patient group analyzed here.

**Figure 5.**
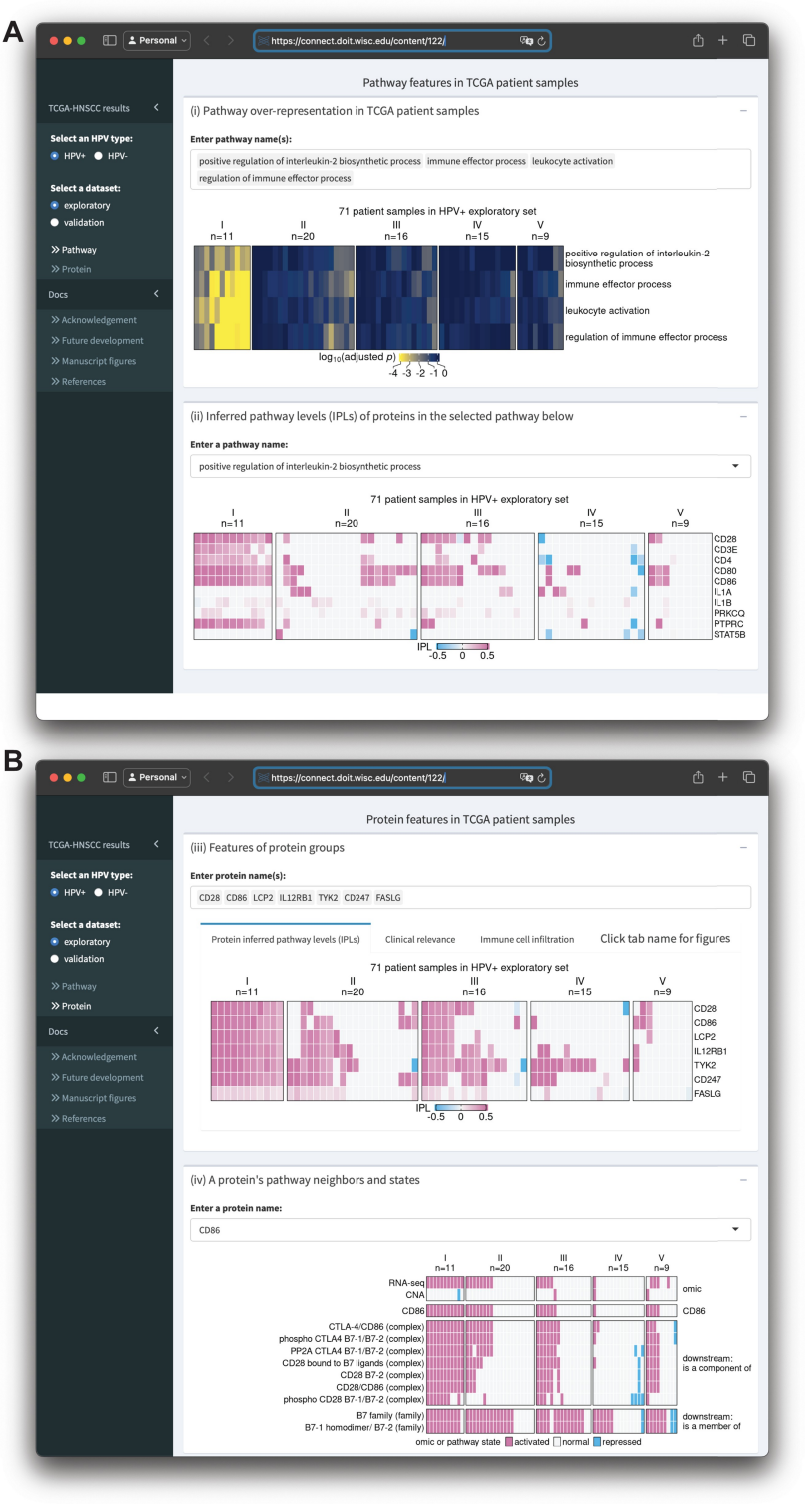
Screenshots of an R Shiny app displaying MPAC results from the HPV+ exploratory set. (A) The upper box shows enrichment of multiple user-selected pathways and the lower box shows protein IPLs from a user-selected pathway. (B) The upper box shows IPLs of multiple user-selected proteins and the lower box shows the pathway states of a user-selected protein and its pathway neighbors as well as its CNA and RNA state.

To understand what factors determine pathway levels of these seven proteins, we developed an approach for pathway state visualization in MPAC. We transformed the continuous-valued inferred pathway levels to discretized pathway states with the values activated, normal, or repressed. The resulting plots presented a protein’s direct pathway network interaction partners and all associated pathway state information for that protein and its partners under the reasoning that a determinant of a functionally implicated protein’s pathway state would have correlated states across all patients (Note 4 and Figs 5–7, available as supplementary data at *Bioinformatics* online). In one patient, TCGA-CR-6487, CD86’s CNA state is repressed, and its RNA state is activated (Fig. 3C). If one were to study CD86 from individual genomic datasets without any pathway information, it would be hard to determine CD86’s functional protein state, illustrating the advantages of our pathway-based approach. Another feature of MPAC’s visualization function shows patient-to-patient variations on pathway determinants. FASLG, for instance, had many upstream and downstream neighbors, of which only two upstream and one downstream complex had pathway states correlated with FASLG, while all the other neighbors had various states across the eleven patients (Figs 7B and 8, available as supplementary data at *Bioinformatics* online). Such diverse states of FASLG’s neighbors likely reflected subtle cancer mechanism differences within this patient group and MPAC can highlight these differences.

To examine potential clinical implications of these seven proteins, we evaluated their association with the patients’ overall survival. For proteins from the same submodule, we used their inferred pathway levels to divide the 71 HPV+ exploratory patients into two groups: those with all relevant proteins in activated pathway levels and those that were not. This approach facilitates easy visualization to examine the association with survival data, while also taking advantage of the fact that all seven proteins exhibit activated pathway states in group I patients. The overall survival distributions of patients from the two groups were compared and evaluated by a log-rank test. For every submodule, although the improvement was not always statistically significant, the set of patients with proteins with activated pathway levels always had a better survival distribution than the set that did not (Fig. 9A, available as supplementary data at *Bioinformatics* online). In particular, when patients had activated pathway levels for both CD247 and FASLG, their overall survival was significantly better than those that did not (log-rank *P *= .00098). Moreover, dividing the same set of patients by the activation of all seven proteins (Fig. 3D) or individual proteins (Fig. 9B, available as supplementary data at *Bioinformatics* online) also produced overall survival advantages in all cases but with log-rank *P* ranging from .0033 to .17. Similar analysis revealed the same trend using the measure of progression-free survival, where patients with the activation of the seven proteins often experienced reduced tumor progression, although the association is not as strong as that observed with overall survival in terms of log-rank *P* (Fig. 10, available as supplementary data at *Bioinformatics* online). Notably, TYK2 and IL12RB1 have been identified by others as associating with HNSCC patient prognosis (Chen *et al.* 2022; He *et al.* 2022). CD28, CD86, CD247, and FASLG have previously been shown to be part of small gene sets that are prognosis-related in HNSCC patients (de Vos *et al.* 2020; Chi *et al.* 2022; Wang *et al.* 2022; Zhu *et al.* 2022). The good association with patient overall survival and reduced tumor progression indicated potential clinical implications of these seven proteins individually and collectively.

Since the seven implicated proteins were identified from the immune response patient group, we explored the relationship between these proteins and immune response. We used a bulk RNA-seq deconvolution method, CIBERSORT in absolute mode (Newman *et al.* 2015), to estimate immune cell composition for the 71 HPV+ exploratory patients and associate them with inferred pathway levels of the seven proteins. CIBERSORT-inferred cell composition was comparable across cell types within the same patient as well as across patients for the same cell type. As in the survival analysis shown in Fig. 3D, patients were stratified by whether they had all seven proteins with activated pathway levels. For patients in this “activated group,” the tumor sample always had substantially higher compositions of T follicular helper cells, CD8+ T cells, regulatory T cells, and M1 and M2 macrophages (Fig. 3E; Fig. 11A, available as supplementary data at *Bioinformatics* online). Thus, similar to the prior results cited above, this association indicates that patients with the seven proteins with activated pathway levels usually had higher levels of immune cell infiltration and further suggests that inferred pathway levels of the seven proteins can serve as indicators for immune infiltration.

### 3.3 Independent validation set confirmed MPAC’s immune response group and key proteins

We used the independent validation set of eighteen HPV+ patients that was held out during MPAC model development and exploratory set analysis to further assess the generality of the seven key proteins identified from the immune response patient group. Thus, we repeated the same MPAC analysis on this validation set, splitting the 18 validation set patients into two groups. The six patients in the resulting validation group II also had many significantly enriched GO terms related to immune response (Fig. 4A). None of the patients with the originally implicated submodule proteins with activated pathway levels died in the interval of record, a notably better overall survival record than those with submodule proteins in normal or repressed pathway levels (Fig. 12A, available as supplementary data at *Bioinformatics* online). Similarly, the overall survival rate of the three patients with all seven proteins in activated pathway levels was always better than the other fifteen patients in the validation set (Fig. 4B). The same trend was observed when stratifying patients by individual proteins (Fig. 12B, available as supplementary data at *Bioinformatics* online). The lack of statistically significant differences between two patient groups was due to the small number of patients (Fig. 4B; Fig. 12, available as supplementary data at *Bioinformatics* online).

We further examined if the activated pathway levels of the seven proteins are also associated with immune cell infiltration, using the same analysis as for the exploratory set. Validation set patients with the seven proteins in activated pathway levels often had higher fractions of T follicular helper cells, CD8+ T cells, regulatory T cells, M1 and M2 macrophages (Fig. 4C), just as we observed in the exploratory set, although the difference was not statistically significant most likely due to the small sample size (Fig. 11B, available as supplementary data at *Bioinformatics* online). Altogether, the independent validation set supported MPAC’s predictions in the exploratory set and greatly reduced the possibility of bias from using the exploratory set alone.

### 3.4 MPAC’s advantages over PARADIGM

MPAC makes multiple improvements (Table 2, available as supplementary data at *Bioinformatics* online) over PARADIGM (Vaske *et al.* 2010), which MPAC runs as a subroutine in Steps 2 and 3 of its workflow. PARADIGM simply divides all the genes into three states with an arbitrarily equal number of entries in each state. In contrast, in Step 1, MPAC defines a gene’s RNA state as normal, activated or repressed for each patient by testing the level of the relevant RNA in that tumor sample for a significant increase or decrease (two standard deviations from the mean of a Gaussian distribution, which equate to the commonly used *P *< .05 cutoff) of that RNA’s expression distribution in normal tissue samples. In Steps 3 and 4, MPAC filters pathway entities for significant inferred pathway level differences from randomly permuted input. Although some PARADIGM applications also used permutations, permutations were not implemented as part of the software, nor were their results used for downstream analysis (Vaske *et al.* 2010; Sedgewick *et al.* 2013). In Step 5, MPAC focuses on the largest patient-specific pathway network subset. This improvement removed from consideration entities in tiny pathways, which were assumed to have less impact on patient pathway alterations and may contribute more noise than signal when predicting patient groups. In Steps 6–8, MPAC provides downstream analysis functions to define patient pathway alterations, predict patient groups, and identify key proteins with potential clinical implications. MPAC is available as an R package on Bioconductor (https://bioconductor.org/packages/MPAC) to streamline the whole process from preparing the omic input data to identifying key proteins for a patient group. We evaluated PARADIGM on the HNSCC dataset and found it was unable to recover the immune response patient group MPAC identified (Note 5 and Fig. 13, available as supplementary data at *Bioinformatics* online).

### 3.5 MPAC robustness analyses

We modified MPAC’s input data to assess the robustness of its outputs. The immune response group can be detected when using 30% or 50% of the samples, but not with 10% (Note 6 and Fig. 14, available as supplementary data at *Bioinformatics* online). Additionally, MPAC demonstrated robustness to variations in the division of exploratory and validation sets (Note 7 and Fig. 15, available as supplementary data at *Bioinformatics* online).

Next, we examined how changing MPAC settings affects the results. The default 100 permutations are needed when the sample size is as small as the 18 patients in the HPV- validation set, but it can be reduced to 50 or 20 for a larger sample size like the 71 patients in the exploratory set (Note 8 and Fig. 16, available as supplementary data at *Bioinformatics* online). The default two standard deviation threshold to define input RNA states can be increased to three, but decreasing to one resulted in largely different results (Note 9 and Fig. 17, available as supplementary data at *Bioinformatics* online). Including more patient-specific pathway sub-networks in MPAC’s Step 5 is unlikely to affect the results, because the second largest sub-networks are much smaller than the largest one (Note 10 and Fig. 18, available as supplementary data at *Bioinformatics* online). Moreover, such an approach does not bias the output toward ubiquitously overrepresented GO terms (Figs 2 and 4A). Integrating CNA with RNA-seq data boosted MPAC performance, because CNA has a stronger impact than RNA-seq on determining a protein’s pathway state overall (Note 11 and Fig. 19, available as supplementary data at *Bioinformatics* online). Separating HPV+ and HPV- patients before applying MPAC is recommended when the input pathway file has little information on HPV-specific pathways (Note 12 and Fig. 20, available as supplementary data at *Bioinformatics* online).

Lastly, we applied MPAC to a different TCGA cancer type to evaluate its generalizability. We chose cholangiocarcinoma because the original study on this cohort (Farshidfar *et al.* 2017) reported pathway analysis results only from bulk RNA-seq data, not multi-omic data. MPAC’s result on the 35 cholangiocarcinoma samples that have both CNA and RNA-seq data shows three groups with distinct biological functions (Note 13 and Fig. 21, available as supplementary data at *Bioinformatics* online), illustrating MPAC’s applicability beyond HNSCC.

### 3.6 An interactive MPAC shiny app supports visualization of results and new analyses

We built an R Shiny app (https://github.com/pliu55/MPAC_Shiny) to display all the HNSCC results generated from this work and support new analyses of the data. It shows enrichment results from 2805 pathways, inferred pathway levels of 19 477 pathway entities, CNA and RNA states of 6251 pathway proteins, and overall survival and immune cell compositions of 492 HNSCC patient samples. Moreover, it illustrates a protein’s pathway membership and network neighbors. On the landing page’s sidebar, users can choose one of the four TCGA-HNSCC datasets: HPV+ or HPV− combined with an exploratory or validation set. The MPAC app visualizes results at both pathway- and protein-levels. On the pathway-level page (Fig. 5A), Shiny app Box (i) displays pathway enrichment results like the ones shown in Figs 2 and 4A, and Fig. 1A, available as supplementary data at *Bioinformatics* online. Users can enter any pathway(s) of interest to look at their enrichments in MPAC-defined patient groups. To understand which proteins lead to a pathway enrichment, Box (ii) shows inferred pathway levels of all the proteins from a pathway. For example, in the pathway “positive regulation of interleukin-2 biosynthetic process,” CD28, CD3E, CD4, CD80, CD86, and PTPRC have positive inferred pathway levels in a majority of group I HPV+ exploratory patient samples (Fig. 5A), suggesting they are the determinants resulting in this pathway’s enrichment in group I patients.

In Box (ii), users can enter or select any pathway of interest to examine their proteins’ inferred pathway levels. At the protein-level page (Fig. 5B), Box (iii) contains results for a group of user-specified proteins. It has three tabs displaying proteins’ inferred pathway levels, overall survival and immune cell composition of patients stratified by proteins’ inferred pathway levels. These figures are similar to Figs 3B, D, and E and 4B and C; Figs 9, 11, and 12, available as supplementary data at *Bioinformatics* online, with the flexibility of showing the results for any user-specified protein(s) on any of the four TCGA-HNSCC datasets. Box (iv) shows a heatmap of the CNA, RNA, and pathway states for any user-entered protein as well as pathway states of the protein’s pathway network neighbors. It is similar to Figs 5–7, available as supplementary data at *Bioinformatics* online with the same flexibility as Box (iii). In summary, the MPAC Shiny app provides a convenient way to explore all the results generated from this work, especially those not presented as figures in this manuscript.

## 4 Discussion

We presented MPAC as a computational framework with several unique features compared to other pathway-based multi-omic integration tools (Table 3, available as supplementary data at *Bioinformatics* online). MPAC calculates inferred pathway levels, predicts patient groups with biologically meaningful pathway profiles, and identifies key proteins with potential clinical associations. One group of HNSCC patients was predicted to have alterations in immune response pathways. This group could not be identified from CNA or RNA-seq data alone or by PARADIGM. This finding illustrates the advantages of our pathway-based multi-omic approach. MPAC can use prior knowledge of pathway interactions in the form of a pathway network to integrate CNA and RNA-seq data and infer proteins’ pathway behavior. A protein’s pathway behavior from MPAC will not solely be inferred from its CNA or RNA but also its pathway neighbors (Fig. 3C; Figs 5–8, available as supplementary data at *Bioinformatics* online). Analysis based on CNA and RNA-seq data alone would miss this important biological principle.

Our analysis showed that MPAC can predict patient groups with potentially relevant clinical properties by their pathway profiles. The results presented above (Fig. 2 and Fig. 1, available as supplementary data at *Bioinformatics* online) identified an immune response patient group in both HPV+ and HPV- HNSCC, which echoes a new subtype defined by recent studies. One study (Huang *et al.* 2021) applied a proteogenomic approach on 108 HPV- HNSCC patients. By considering CNA, RNA, miRNA, protein, and phosphopeptide data, the authors defined three subtypes of HNSCC: chromosome instability, basal, and immune. These subtypes mirrored the immune response patient groups MPAC identified in both HPV+ and HPV− patients. Moreover, the authors analyzed immune-hot tumors and revealed the presence of both cytotoxic immune cells (e.g. CD8+ T cells and M1 macrophages) and immunosuppressive cells (e.g. regulatory T cells and M2 macrophages). This is consistent with our analysis of HPV+ tumors stratified by inferred pathway levels of the seven proteins identified from the immune response patient group. Tumor samples with activated pathway levels of any of the seven proteins always had a higher fraction of CD8+ T cells, regulatory T cells, and M1 and M2 macrophages in both the exploratory and validation sets (Figs 3E and 4C). Second, a multi-omic analysis of 33 TCGA cancer types (Tiong *et al.* 2022) identified gene groups enriched by immune response as well as cell cycle, which were also observed in MPAC’s results (Figs 2 and 4A; Fig. 1A, available as supplementary data at *Bioinformatics* online). The agreement between these two studies supports MPAC’s discovery of the immune response patient group.

Further, not only did the seven proteins identified by MPAC associate with immune cell composition, but their activated pathway levels also associated with better overall survival. This was demonstrated in our exploratory patient set (Fig. 3D and Fig. 9, available as supplementary data at *Bioinformatics* online) and supported by our validation set (Fig. 4B and Fig. 12, available as supplementary data at *Bioinformatics* online). The corroboration by the validation set illustrates a major strength of MPAC. To understand these seven proteins’ clinical values and whether they could serve as biomarkers would require a prospective patient cohort, which is not available to us currently [as of 6 May 2024, according to cBioportal (de Bruijn *et al.* 2023) the only HNSCC dataset that has both CNA and RNA-seq is the TCGA dataset used in this work]. However, the analyses here demonstrated how the MPAC software could be applied in a prospective setting.

MPAC has several advantages over the PARADIGM algorithm that it calls as a subroutine. When preparing the input for predicting pathway levels, MPAC uses a data-driven approach to define each gene’s discrete states based on both tumor and normal tissue samples, whereas PARADIGM arbitrarily assigns the top, middle and lower third of omic-ranked genes as activated, normal and repressed (Vaske *et al.* 2010). MPAC also provides downstream analyses on inferred pathway levels, including built-in permutation testing, defining altered pathways, predicting patient groups, and identifying key proteins with potential clinical implications. All these functions have been implemented in an R package available through Bioconductor making it easier for others to use in their studies. The MPAC R Shiny app also supports convenient visualizations of the MPAC predictions.

Multi-omic integration methods have been developed for diverse applications (Maghsoudi *et al.* 2022; Zitnik *et al.* 2024), such as embedding single-cell data (Argelaguet *et al.* 2020; Ashuach *et al.* 2023), clustering cancer samples (Wang *et al.* 2014; Chauvel *et al.* 2020), and pathway reconstruction (Paull *et al.* 2013; Tuncbag *et al.* 2016; Winkler *et al.* 2022). Multi-omics analyses have been particularly prominent in cancer, with pathway enrichment (Paczkowska *et al.* 2020), representation learning (Leng *et al.* 2022), supervised prediction of cancer subtypes or patient outcomes (Poirion *et al.* 2021; Choi and Chae, 2023), and biologically interpretable neural networks (Wysocka *et al.* 2023) as representative areas of study. MPAC’s unique role in this methodological landscape is that through PARADIGM it directly uses pathway interactions to combine information across omic data types, learn protein activities, and conduct downstream analysis with those protein activities.

In this work, we limited the input multi-omic data to CNA and RNA-seq, given PARADIGM’s previous success with these two data types. With the availability of many other types of omic data from TCGA and the Clinical Proteomic Tumor Analysis Consortium (Huang *et al.* 2021) on large cohorts of cancer patients, time course multi-omic data (Stein-O’Brien *et al.* 2018a), single-cell RNA-seq (Puram *et al.* 2017), spatial transcriptomics (Lee *et al.* 2024; Li *et al.* 2024), and spatial proteomics (Causer *et al.* 2023), one of our future goals is to make MPAC compatible with as many omic data types as possible. This requires extending the MPAC software as well as the input biological pathways to include knowledge on the relevant molecules and associated regulatory mechanisms. Expanding the input biological pathways will also help disease-specific studies as shown in our analysis on separating HPV+ and HPV- HNSCC patient samples (Note 12 and Fig. 20, available as supplementary data at *Bioinformatics* online). For studies focusing on a specific disease or condition, smaller and pertinent input pathways will expedite MPAC’s PARADIGM subroutine calculations on permuted data (Note 14 and Fig. 22, available as supplementary data at *Bioinformatics* online).

## Supplementary Material

btaf490_Supplementary_Data

## Data Availability

The MPAC package is available at Bioconductor (https://bioconductor.org/packages/MPAC) and archived on Zenodo (https://doi.org/10.5281/zenodo.10805479). MPAC’s Shiny app is accessible at https://connect.doit.wisc.edu/content/122/. The source code for MPAC’s Shiny app is available at GitHub (https://github.com/pliu55/MPAC_Shiny) and archived on Zenodo (https://doi.org/10.5281/zenodo.11623974). Both the R package and Shiny app are available under the GPL-3.0 license.
