# Supplementary material for: MPAC: a computational framework for inferring pathway activities from multi-omic data
Source: Bioinformatics. 2025 Sep 11;41(10):btaf490. doi: 10.1093/bioinformatics/btaf490 (PMC12496133; doi:10.1093/bioinformatics/btaf490)
Supplement: btaf490_Supplementary_Data [file btaf490_supplementary_data.pdf]

Supplementary Information

MPAC: a computational framework for inferring pathway activities from multi-omic data

Peng Liu, David Page, Paul Ahlquist, Irene M. Ong, Anthony Gitter

Table of Contents

Supplementary Notes 4
Supplementary Note 1. Pseudocode of MPAC 4
Supplementary Note 2. MPAC method details 5
Supplementary Note 3. MPAC groups cannot be recovered by individual omic data types alone . 5
Supplementary Note 4. Determinants of CD86's pathway states 6
Supplementary Note 5. PARADIGM cannot recover MPAC's immune response patient group . . 6
Supplementary Note 6. Subsampling analysis of MPAC 7
Supplementary Note 7. MPAC is robust to variations in exploratory and validation set splits . . . 7
Supplementary Note 8. Required number of permutations in MPAC 7
Supplementary Note 9. Number of standard deviation threshold to define input RNA states . . . 8
Supplementary Note 10. The largest pathway sub-networks are much bigger than the second largest ones 8
Supplementary Note 11. CNA has a stronger impact than RNA-seq in determining protein pathway states 9
Supplementary Note 12. MPAC cannot separate HPV+ and HPV- patients with insufficient input pathway information 9
Supplementary Note 13. MPAC reveals three biologically distinct cholangiocarcinoma patient groups 10
Supplementary Note 14. Time and memory requirements for MPAC 10

Supplementary Tables 11
Supplementary Table 1. Number of patient samples in each dataset 11
Supplementary Table 2. Comparison of MPAC and PARADIGM features 12
Supplementary Table 3. Feature comparison of MPAC with other related software 13

Supplementary Figures 14

List of Supplementary Figures

1 HPV- exploratory set patient samples grouped by GO term enrichment based on IPL (A), CNA (B), or RNA (C) . . . 14
2 HPV+ exploratory set patient samples grouped by GO term enrichment based on CNA (A) or RNA (B) . . . 15
3 HPV+ exploratory set patient samples grouped by MPAC's input RNA states . . . 16
4 HPV+ exploratory set patient samples grouped by MPAC's input CNA states . . . 17
5 CNA, RNA, and pathway states of CD28 (A), CD86 (B), and LCP2 (C), as well as pathway states of their pathway network neighbors in the eleven group I patients . . . 18
6 CNA, RNA, and pathway states of TYK2 (A) and IL12RB1 (B), as well as pathway states of their pathway network neighbors in the eleven group I patients . . . 19
7 CNA, RNA, and pathway states of CD247 (A) and FASLG (B), as well as pathway states of their pathway network neighbors in the eleven group I patients . . . 20
8 CNA, RNA, and pathway states of FASLG as well as pathway states of its pathway network neighbors in a group I patient TCGA-CR-7385 . . . 21
9 Overall survival of HPV+ exploratory set patient samples stratified by the inferred pathway levels (IPLs) of proteins from the same submodule (A) or individual protein (B) . . . 22
10 Progression-free survival of HPV+ exploratory set patient samples stratified by the inferred pathway levels (IPLs) of all the seven proteins combined (A); proteins from the same submodule (B), or individual protein (C) . . . 23
11 Immune cell compositions stratified by the inferred pathway levels (IPLs) of all the seven proteins combined for HPV+ exploratory (A) and validation (B) set . . . 24
12 Overall survival of HPV+ validation set patient samples stratified by the inferred pathway levels (IPLs) of proteins from the same submodule (A) or individual protein (B) . . . 25
13 Clustering patient samples and annotating patient groups by PARADIGM IPLs from Pan-Cancer Atlas . . . 26
14 Subsampling analysis by MPAC on HPV+ patient samples . . . 28
15 Exploratory and validation set resampling of MPAC on HPV+ patient samples . . . 32
16 Distribution of immune response patient samples under different numbers of permutations for HPV+ exploratory (A) and validation (B) set . . . 33
17 Impact of one, two, or three standard deviation thresholds on defining input RNA states for HPV+ validation set . . . 35
18 Comparison of the sizes of the largest and the second largest sub-network for HPV+ exploratory (A) and validation (B) set . . . 36
19 Comparison of the number of proteins that have their pathway states agreed with CNA and/or RNA-seq data . . . 37
20 MPAC cannot separate HPV+ and HPV- samples due to insufficient input pathway knowledge 38
21 Patient sample groups and significantly overrepresented GO terms for the TCGA cholangiocarcinoma cohort . . . 39
22 Time (A) and memory (B) usage by MPAC's PARADIGM subroutine runs on real and permuted data from the 71 HPV+ exploratory set patient samples . . . 40

Supplementary Notes

Supplementary Note 1. Pseudocode of MPAC

```
FOR each sample:
    ## Step 1
    prepare CNA states from real data

    prepare RNA states from real data

    ## Step 2
    compute IPLs by PARADIGM using CNA and RNA states from real data as well
    as the input pathway definition

    ## Step 3
    REPEAT 100 times:
        randomly permute CNA and RNA states between genes

        compute IPLs by PARADIGM using CNA and RNA states from permuted data as
        well as the input pathway definition

    ## Step 4
    FOR each pathway entity:
        filter entity's IPL from real data by its 100 IPLs from permuted data

    ## Step 5
    based on the input pathway definition, select the largest sub-pathway with
    all of its entities with non-zero IPLs

    ## Step 6
    FOR each GO term in GMT file collections:
        do enrichment analysis using genes from the largest sub-pathway

cluster samples by their GO enrichment results

## Step 7
pick a sample group of interest

find submodules shared by the largest sub-pathways of all samples in this group

identify key pathway proteins from submodules

## Step 8
evaluate the association of key protein’s IPLs with patients’ clinical data
```

Supplementary Note 2. MPAC method details

A clustering method originally designed for single-cell RNA-seq analysis was adapted, where a patient tumor sample was treated as a cell and the $|\log_{10}(\text{adjusted } p)|$ was treated as a gene's expression level. Gene variance was modeled by the `modelGeneVar()` function from the scran package (Lun et al., 2016) (version 1.20.1), and the top 100 genes were selected. Patients were grouped by the Louvain method from the igraph R package (version 1.2.11) with 10 or 20 nearest neighbors for HPV+ or HPV-, respectively. Patient groups do not necessarily have similar sizes (Figure 2; Figure 4A; Supplementary Figure 21).

Changes in the igraph R package starting with version 1.3 affected the reproducibility of our results but not the main conclusions from our analyses. Starting in igraph version 1.3, the Louvain method is no longer deterministic and different runs may generate different clustering results (https://github.com/igraph/rigraph/issues/539). To evaluate the impact of non-deterministic clustering, we executed three independent batches of 10,000 random Louvain clustering runs. We used the Adjusted Rand Index (ARI) to measure the difference between groups from these random runs with the original groups from igraph version 1.2.11. The median ARIs were 0.92, 1.00, and 0.78 for the HPV+ exploratory set, HPV+ validation set, and HPV- exploratory set, respectively, (HPV- validation set groups were not used in this analysis), indicating small variations in the patient cluster membership. The 11- and 6-patient immune groups in the HPV+ exploratory and validation set were largely unchanged (identical in >9,200 and >8,800 out of 10,000 runs, respectively). For the HPV- exploratory set, the top five grouping results in each batch have either 30- or 32-patient groups and both groups are enriched with immune response GO terms. In summary, despite the randomness introduced in igraph version 1.3, our findings are still maintained.

The most time-consuming part of MPAC is running PARADIGM using the Pan-Cancer Atlas pathways on real and permuted data. It took a maximum of 4 days to run on real data and two weeks on permuted data using 500~600 MB memory on each sample (Supplementary Note 14; Supplementary Figure 22).

To summarize pathway features for a group, we plotted heatmaps of $\log_{10}(\text{adjusted } p)$ values for GO terms with adjusted $p < 0.05$ in 100% of the patients from the same group (e.g., Figure 2 and Supplementary Figure 1A). When very few GO terms met this criterion, we lowered the percentage threshold (e.g., $\geq$ 80% in Figure 4A, $\geq$ 60% in Supplementary Figure 1C) or by specifying a minimum number (e.g., $\geq$ 3 in Supplementary Figure 1B) of patients in order to include more GO terms.

MPAC offers several functions for users to explore the networks generated during the analysis. The largest network subset can be obtained by MPAC's `subNtw()` function, while consensus pathway submodules can be obtained by MPAC's `conMtf()` function and visualized by MPAC's `pltConMtf()` function.

For survival analysis, we used the inferred pathway levels of one or multiple proteins to stratify patients into two groups: those with all the protein(s) in activated pathway states (i.e., positive inferred pathway level values) and those not. A log-rank test p-value was calculated to compare the survival distribution of the two groups.

Supplementary Note 3. MPAC groups cannot be recovered by individual omic data types alone

MPAC predicted patient groups with distinct pathway profiles by integrating multi-omic data. We found that the same groups and pathways cannot be found by examining individual omic data types alone. Starting

from either CNA or RNA-seq data, we conducted two tests: one performing GO enrichment on each single omic data type and then grouping enriched GO terms, like the MPAC workflow, and the other by grouping patients via their single omic data first and then finding commonly enriched GO terms within each group. In the first test, HPV+ patients' CNA data had very few GO terms enriched, and even these were only enriched in a small number of patients (Supplementary Figure 2A). As a result, no group prediction could be made. RNA-seq data was more informative than CNA data, and four patient groups could be predicted (Supplementary Figure 2B). Groups III and IV were related to cell cycle and morphogenesis pathways, respectively, both of which had also been observed in the MPAC results. The immune response patient group predicted by MPAC, however, was not observed from CNA or RNA-seq data, indicating a unique insight from MPAC. For HPV- patients, CNA data did not lead to any patient groups due to insufficient significantly enriched GO terms (Supplementary Figure 1B). RNA-seq data led to six groups. Two of them were related to cell cycle and immune response (Supplementary Figure 1C), which were also observed in MPAC results.

To demonstrate the robustness of this result, we performed another test by grouping patients first and followed by GO enrichment. We applied K-means clustering to the RNA-seq data and divided HPV+ patients into two to six groups. The cluster membership remained stable under different numbers of groups (Supplementary Figure 3A). Therefore, we used five groups (Supplementary Figure 3B) for GO enrichment analysis so that every group had at least two samples while maintaining as many groups as possible. Groups I, II, IV, and V did not have any top GO terms related to immune response (Supplementary Figure 3 C-D and F-G). Group III was predominantly enriched with cell cycle-related GO terms with only one GO term (lymphocyte activation) related to immune response (Supplementary Figure 3E). Moreover, this single term was less consistently enriched than the >20 immune response GO terms from MPAC (Figure 2A). We performed the same analysis on CNA data. Stable grouping membership was observed (Supplementary Figure 4A), and the three-groups result (Supplementary Figure 4B) was used for GO enrichment analysis. No GO term was significantly overrepresented in at least half of the samples in any group. In summary, by jointly modeling both CNA and RNA-seq data, MPAC identified a large and unique HPV+ patient group related to immune response that could not be recovered from either individual omic data type alone.

Supplementary Note 4. Determinants of CD86's pathway states

In all the eleven Group I patients, CD86's pathway states agreed with its RNA states as well as with six of its seven downstream interacting complexes (Supplementary Figure 5B), indicating their parallel roles in determining CD86's pathway states. In contrast, CD86's two downstream gene families and one downstream complex had states that disagreed with CD86 in one or three patients, respectively (Supplementary Figure 5B), suggesting a less influential role. CD86's CNA states, to the other extreme, did not agree with CD86's pathway state in any patient (Supplementary Figure 5B).

Supplementary Note 5. PARADIGM cannot recover MPAC's immune response patient group

To assess MPAC's benefits over PARADIGM, we asked whether PARADIGM can also discover an immune response patient group as MPAC does. We downloaded PARADIGM's inferred pathway levels from NCI's PanCanAtlas website. 70 of the 71 patients in the HPV+ exploratory set and all 18 patients in the validation

set have PARADIGM inferred pathway levels available. We applied Gene Set Enrichment Analysis (GSEA) on the same set of GO terms used by MPAC and then clustered patients by K-means based on the GSEA results. For the HPV+ exploratory set, we tried for two to five groups, and two groups appeared to be a good choice because it does not have any group with just one or two samples (Supplementary Figure 13A). Only two GO terms are significantly overrepresented in $\geq$ 50% of samples in Group c2 and none for Group c1 (Supplementary Figure 13B). The two GO terms are related to development, which is similar to Group V by MPAC (Figure 2). PARADIGM does not find an immune response group. For the HPV+ validation set, we also tried two to five groups, and two groups were taken for the same reason as for the exploratory set (Supplementary Figure 13C). Six GO terms are significantly overrepresented in $\geq$ 80% of samples of Group c2 and none for Group c1 (Supplementary Figure 13D). All the six GO terms are related to development of morphogenesis. Once again, PARADIGM does not find an immune response group. In summary, MPAC shows advantages over PARADIGM because MPAC recovers a unique immune response patient group and PARADIGM's largest clusters in both the HPV+ exploratory and validation sets are not enriched for any GO terms.

Supplementary Note 6. Subsampling analysis of MPAC

We did a subsampling analysis by randomly taking 10%, 30%, and 50% of the 89 HPV+ exploratory and validation sets. We applied MPAC on each of them and found that an immune response patient group can be obtained at both 30% and 50%, but not at 10% (Supplementary Figure 14), indicating MPAC may not generate consistent results for sample size < 10.

Supplementary Note 7. MPAC is robust to variations in exploratory and validation set splits

We created multiple data splits that swapped which of the 89 HPV+ samples were used as the exploratory and validation set, similar to 5-fold cross-validation in supervised learning. The existing HPV+ exploratory (Figure 2) and validation (Figure 4) sets were used as split #1. The same MPAC protocol and parameters were applied to splits #2 to #5, so there is no supervised learning per se on the exploratory sets from these four splits. Most of the results generated an immune response group except for split #2's validation set, split #5's exploratory and validation sets (Supplementary Figure 15). Split #3's validation set has Group c2 as an immune response group and this group also contains significantly overrepresented cell cycle GO terms (Supplementary Figure 15D). Overall, this exploratory and validation set resampling result demonstrated the robustness of MPAC.

Supplementary Note 8. Required number of permutations in MPAC

To find out the number of permutations required for a valid result, we randomly selected 10, 20, and 50 permutations from the existing 100 permutations for each HPV+ sample. With them, we ran MPAC under the same protocol. We checked if the 11 and 6 immune response samples from the exploratory and validation set, respectively, were still grouped together and with no other samples added under a smaller number of permutations. For the exploratory set, when using 50 or 20 permutations, 9 or 10 of the

11 immune response samples continued to fall into the same group with at most 1 other sample added (Supplementary Figure 16A). When using 10 permutations, although 10 immune response samples were clustered together, 6 other samples came to the same group (Supplementary Figure 16A) and thus added more noise to the biological function of this group. For the validation set, the 6 immune response samples were spread almost evenly into different groups with at most 3 or 4 samples in a group with 2 or more other samples added by 50, 20, or 10 permutations (Supplementary Figure 16B). This analysis suggests that if a user has about 70 samples like the HPV+ exploratory set, 50 or 20 permutations would be ok. If a user has about 20 samples like the HPV+ validation set, 100 permutations would be required. In the MPAC package, we set the default to 100 and provide an option for users to adjust this number based on their sample sizes.

Supplementary Note 9. Number of standard deviation threshold to define input RNA states

The reason MPAC takes two standard deviations (SD) as the threshold is because it corresponds to the commonly used $p < 0.05$ cutoff. For any gene, MPAC extracts its expression levels from RNA-seq data of normal samples and fits them by a Gaussian distribution. Two-SD threshold on such a Gaussian distribution is at a $p = 0.05$ cutoff (three SD is at a $p = 0.01$ cutoff and one SD is at a $p = 0.32$ cutoff). To evaluate the impact by different SD, we tried 1, 2, and 3 SD on the 18 samples in the HPV+ validation set. We first checked their effects on the RNA states of all the genes. Those from 2 and 3 SD are similar to each other, especially for repressed and normal states (Supplementary Figure 17A), while those from 1 SD are largely different from those of 2 or 3 SD. Next, we compared the sample groups from different SD. Groups from 2 and 3 SD share good overlaps with three Group II samples in common (Supplementary Figure 17B), whereas groups from 1 SD are totally different from those from 2 and 3 SD. Such dissimilarity is further supported by the overrepresented pathways for each group. By 1 SD, its Group II has many GO terms related to cell cycle, Wnt signaling, etc, but very few related to immune response (Supplementary Figure 17C). In contrast, by 3 SD, its Group II has a large fraction of overrepresented GO terms related to immune response (Supplementary Figure 17D), which is similar to those by 2 SD (Figure 4A). Taken together, because of the commonly used $p < 0.05$ or < 0.01 cutoff, and because of the agreement on the RNA states and sample groups, we recommend users consider 2 or 3 SD to define input RNA states.

Supplementary Note 10. The largest pathway sub-networks are much bigger than the second largest ones

In MPAC, each sample has its own largest sub-network. The reason why MPAC only considers the largest sub-network is because the second largest sub-network is much smaller compared to the largest one. To illustrate this, we compared the sizes between the two from each sample in terms of two types of entities:

1. all entities in the sub-networks, which include proteins, complexes, families, etc
2. only proteins that have their genes in the GO terms that were used for MPAC's enrichment analysis

In both HPV+ exploratory and validation set, the largest sub-networks are overwhelmingly larger than the second largest sub-networks for both all entities and GO terms genes (Supplementary Figure 18). The

sub-network's 'stability' could be affected by a few factors, such as the number of permutations or the number of standard deviations to define input RNA states.

Supplementary Note 11. CNA has a stronger impact than RNA-seq in determining protein pathway states

MPAC integrates both CNA and RNA-seq data, but CNA's role is not evident from the key proteins shown earlier (Supplementary Figure 5, Supplementary Figure 6, and Supplementary Figure 7). We extended the analysis to all the 6,251 pathway proteins with both CNA and RNA-seq data for all the 89 HPV+ exploratory and validation set samples. The comparison results are divided into the following four categories by whether a pathway state:

- Different from the one of CNA and RNA
- Same as the one of both CNA and RNA
- Same as the one of RNA, but different from the one of CNA
- Same as the one of CNA, but different from the one of RNA

For the majority of the 89 samples, the fraction of proteins that have pathway states only the same as CNA (blue bars in Supplementary Figure 19) is higher than those that have pathway states only the same as RNA (orange bars in Supplementary Figure 19), indicating that CNA has a stronger impact than RNA on determining a protein's pathway state overall. We also note that CNA does not have a stronger impact than RNA in every sample. 10 of the 89 samples have RNA with a stronger impact than CNA.

Supplementary Note 12. MPAC cannot separate HPV+ and HPV- patients with insufficient input pathway information

The reason we split HPV+ and HPV- before applying MPAC is because they are largely different in oncogenic pathways, tumor biology, and clinical treatment response. To test if MPAC can separate HPV+ and HPV-, we applied MPAC on the 393 samples from the HPV+ and HPV- exploratory sets. MPAC gives three groups and every group contains both HPV+ and HPV- samples (Supplementary Figure 20A), indicating that MPAC cannot separate HPV+ and HPV-. This is largely because the input pathways for MPAC have insufficient information on HPV. The pathways are from TCGA Pan-Cancer Atlas and are not designed specifically for HPV. They do not have any information on HPV protein E7 and very little on HPV protein E6. For example, it is well known that HPV E6 represses TP53 in HPV+, but MPAC's pathways do not contain such interaction. To illustrate further, we checked the MPAC IPLs of TP53 as well as another protein CDKN2A, which is known to have alterations predominantly only in HPV-. For both of them, their IPLs do not show a difference between HPV+ and HPV- (Supplementary Figure 20B). Despite there being a statistical difference of CDKN2A IPLs (Wilcoxon $p = 2.26 * 10^{-5}$), CDKN2A and TP53's IPLs are within the same IPL ranges between HPV+ and HPV-, illustrating the impact of insufficient input pathway knowledge on key HPV-specific proteins and the difficulty on separating HPV+ and HPV-. The TCGA HNSCC study does not separate HPV+ and HPV- either using PARADIGM alone. In their paper's Supplementary Figure S7.11 (https://doi.org/10.1038/nature14129), HPV+ samples are mainly in subtype 3 (black), but this subtype also contains a substantial number of HPV- samples.

Supplementary Note 13. MPAC reveals three biologically distinct cholangiocarcinoma patient groups

MPAC's result on the 35 cholangiocarcinoma samples that have both CNA and RNA-seq data shows three groups with distinct biological functions (Supplementary Figure 21). Group c1 is mainly on metabolic processes, especially on xenobiotic metabolic processes. Group c2 is mainly on apoptotic process and response to unfolded protein. Group c3's function is unclear because it does not have any GO term significantly overrepresented in $\geq$ 80% of samples (i.e. $\geq$ 8 samples). These three groups illustrate MPAC's applicability to a cancer type other than HNSCC. The significantly overrepresented GO terms in this cohort are different from those in HNSCC, largely because of different disease mechanisms. Additionally, 74 and 53 submodule proteins were identified in Group c1 and c2, respectively. Because the number of submodule proteins exceeds the small sample size of 35, we did not pursue survival analysis in this cohort.

Supplementary Note 14. Time and memory requirements for MPAC

The major computational bottleneck for MPAC is running PARADIGM using the Pan-Cancer Atlas pathways on the large number of permuted data. For the 71 samples in HPV+ exploratory set, 100 permuted data for each sample resulted in 7,100 PARADIGM runs. This is the main reason why we utilized HTCondor from the Center for High Throughput Computing. Although most jobs on permuted data finished in 3 days, the longest one took about two weeks (Supplementary Figure 22A). In comparison, for jobs on real data, all of them finished in about four days (Supplementary Figure 22A). These jobs' memory usage is not heavy. Only 500 to 600 MB is needed for most of them for both real and permuted data (Supplementary Figure 22B). Jobs on permuted data may require longer time and more memory than on real data because permuted data likely have input omic states that are discordant with the input pathways and thus it is more computationally expensive to find optimal pathway states for both. Lastly, please note that, because of the nature of HTCondor, the aforementioned jobs were run on a heterogeneous resource (e.g., CPU speed, architecture).

Supplementary Tables

Supplementary Table 1. Number of patient samples in each dataset

	HPV+	HPV-
exploratory	71	322
validation	18	81

HNSCC patient samples were stratified first by HPV subtypes and then by a random 80% and 20% split into exploratory and validation set to tune and test MPAC, respectively.

Supplementary Table 2. Comparison of MPAC and PARADIGM features

	MPAC	PARADIGM
Uses RNA-seq from normal samples to define RNA states	✓	✗
Uses prior pathway knowledge as input	✓	✓
Integrates CNA and RNA-seq data	✓	✓
Uses permuted input to identify randomly observed IPLs	✓	✗
Focuses on the largest sample-specific pathway sub-network	✓	✗
Clusters samples by their altered pathways	✓	✗
Defines the biological functions of sample groups	✓	✗
Identifies key proteins shared by samples in a group	✓	✗
Identifies the determinants of a protein's pathway state	✓	✗

A check mark (✓) represents that feature is implemented and a cross mark (✗) indicates the lack of the feature.

Supplementary Table 3. Feature comparison of MPAC with other related software

	MPAC	MOMA	OncoSig	COSMOS
Integrates multi-omic data	✓	✓	✓	✓
Utilizes prior pathway network knowledge as input	✓	✗	✗[¶]	✓
Considers comprehensive molecular interactions at DNA, RNA, and protein level	✓	✗[§]	✗[§]	✗[†]
Interprets result in terms of pathways	✓	✓	✓	✓
Clusters samples by their pathway profiles and characterizes sample group by altered pathways	✓	✓	✗	✗

A check mark (✓) represents that feature is implemented and a cross mark (✗) indicates the lack of the feature.

[¶] OncoSig takes a binary input on whether a protein is in a MSigDB gene set, but no interaction knowledge (i.e., pathway networks) can be used.

[§] MOMA and OncoSig consider direct interactions on master regulators. Indirect interactions of proteins further downstream of master regulators are not included.

[†] COSMOS considers comprehensive molecular interactions over signaling pathways, gene regulations, and metabolic networks, but copy-number alteration (i.e., at DNA level) is not included.

Supplementary Figures

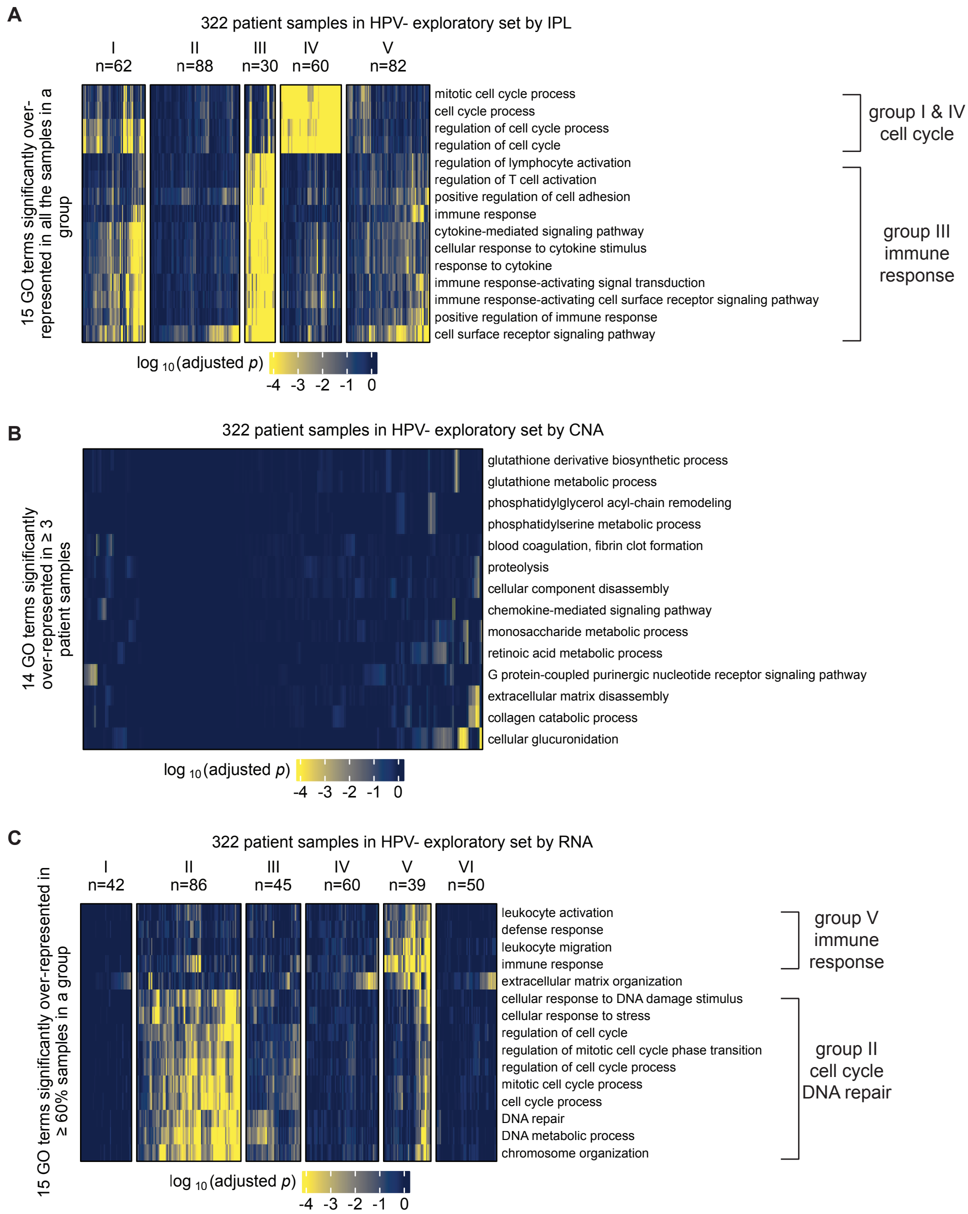

Supplementary Figure 1: HPV- exploratory set patient samples grouped by GO term enrichment based on IPL (A), CNA (B), or RNA (C). The selection threshold was lowered to ≥ 3 (B) or $\geq 60\%$ (C) to include more GO terms to avoid bias.

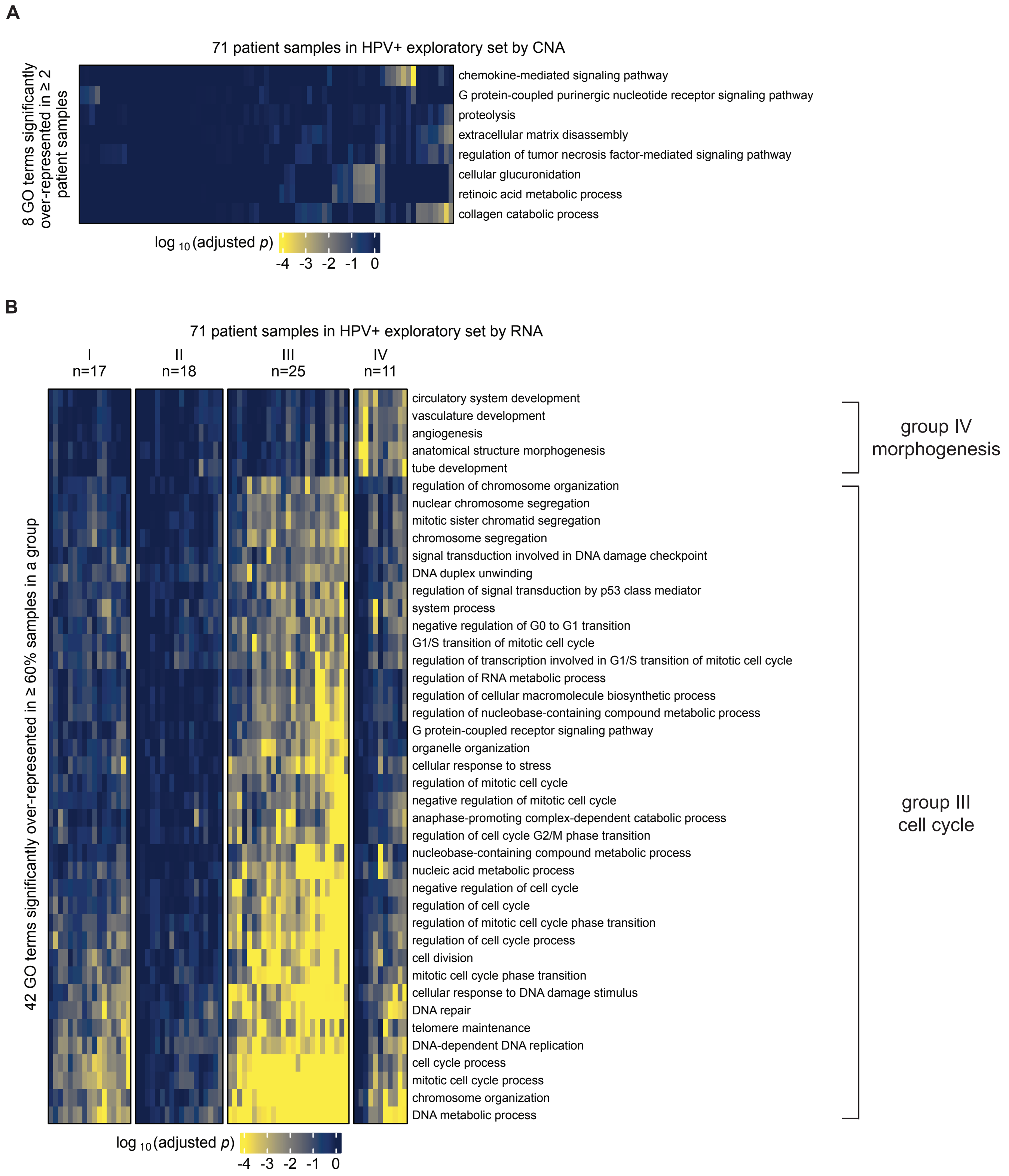

Supplementary Figure 2: HPV+ exploratory set patient samples grouped by GO term enrichment based on CNA (A) or RNA (B). The selection threshold was lowered to ≥ 2 (A) or $\geq 60\%$ (B) to include more GO terms to avoid bias.

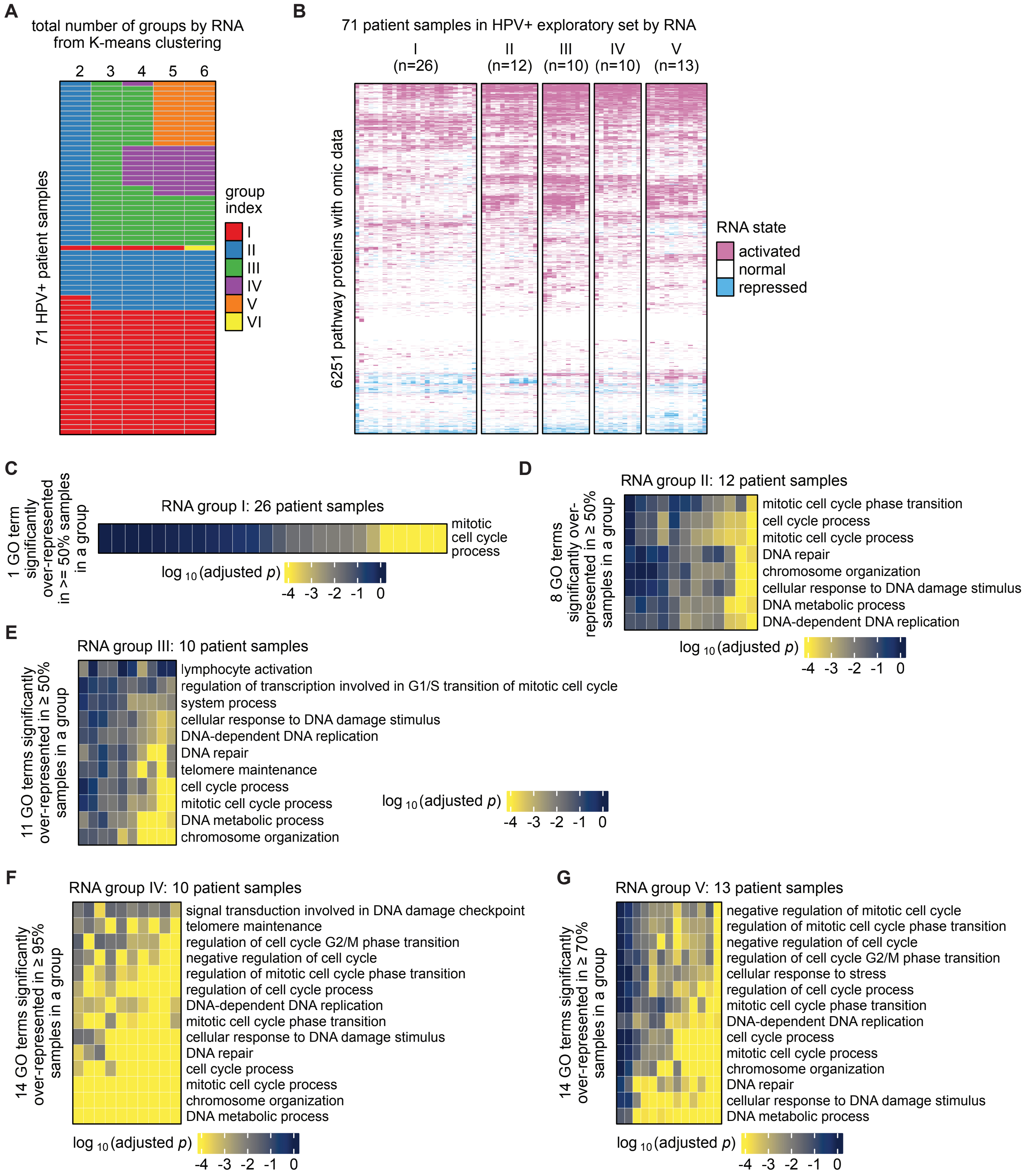

Supplementary Figure 3: HPV+ exploratory set patient samples grouped by MPAC's input RNA states. (A) Clustering results by K-means under a pre-specified total number of groups ranging from two to six; (B) Clustering results by K-means with a pre-specified five groups; (C–G) Top significantly enriched GO terms in group I (C), II (D), III (E), IV (F), and V (G). The selection threshold was lowered to $\geq$ 95% (F), $\geq$ 70% (G), or $\geq$ 50% (C–E) in order to include more GO terms to avoid bias.

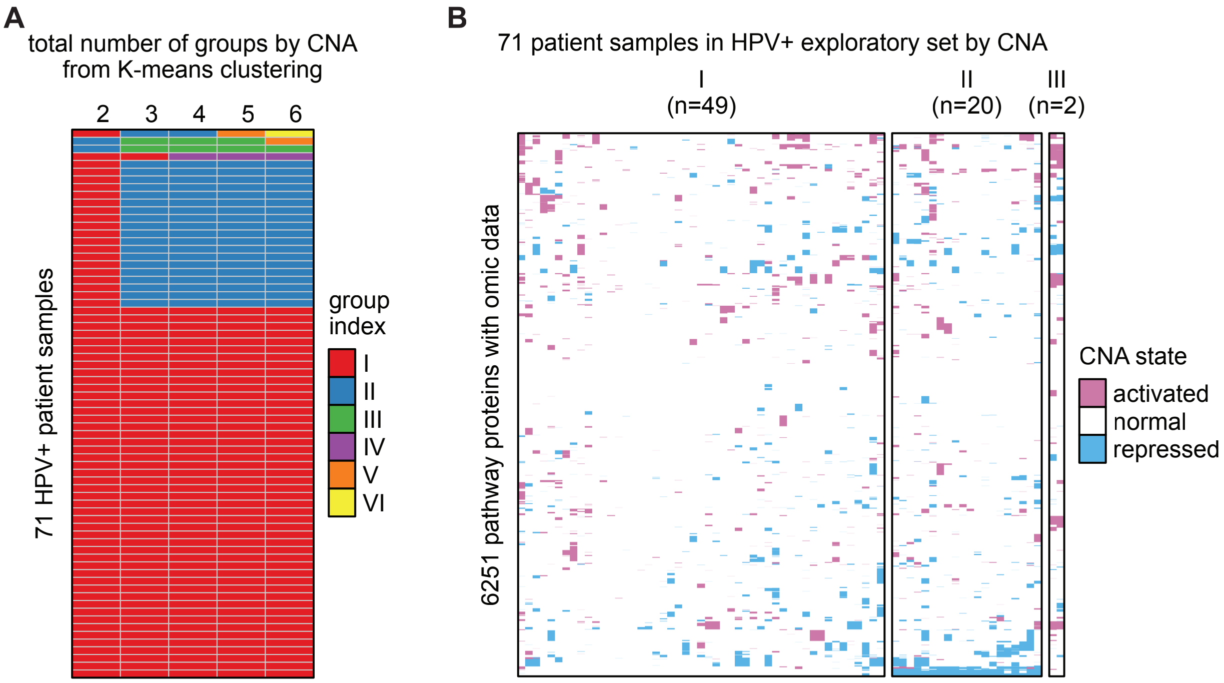

Supplementary Figure 4: HPV+ exploratory set patient samples grouped by MPAC's input CNA states. (A) Clustering results by K-means under a pre-specified total number of groups ranging from two to six; (B) Clustering results by K-means with a pre-specified three groups.

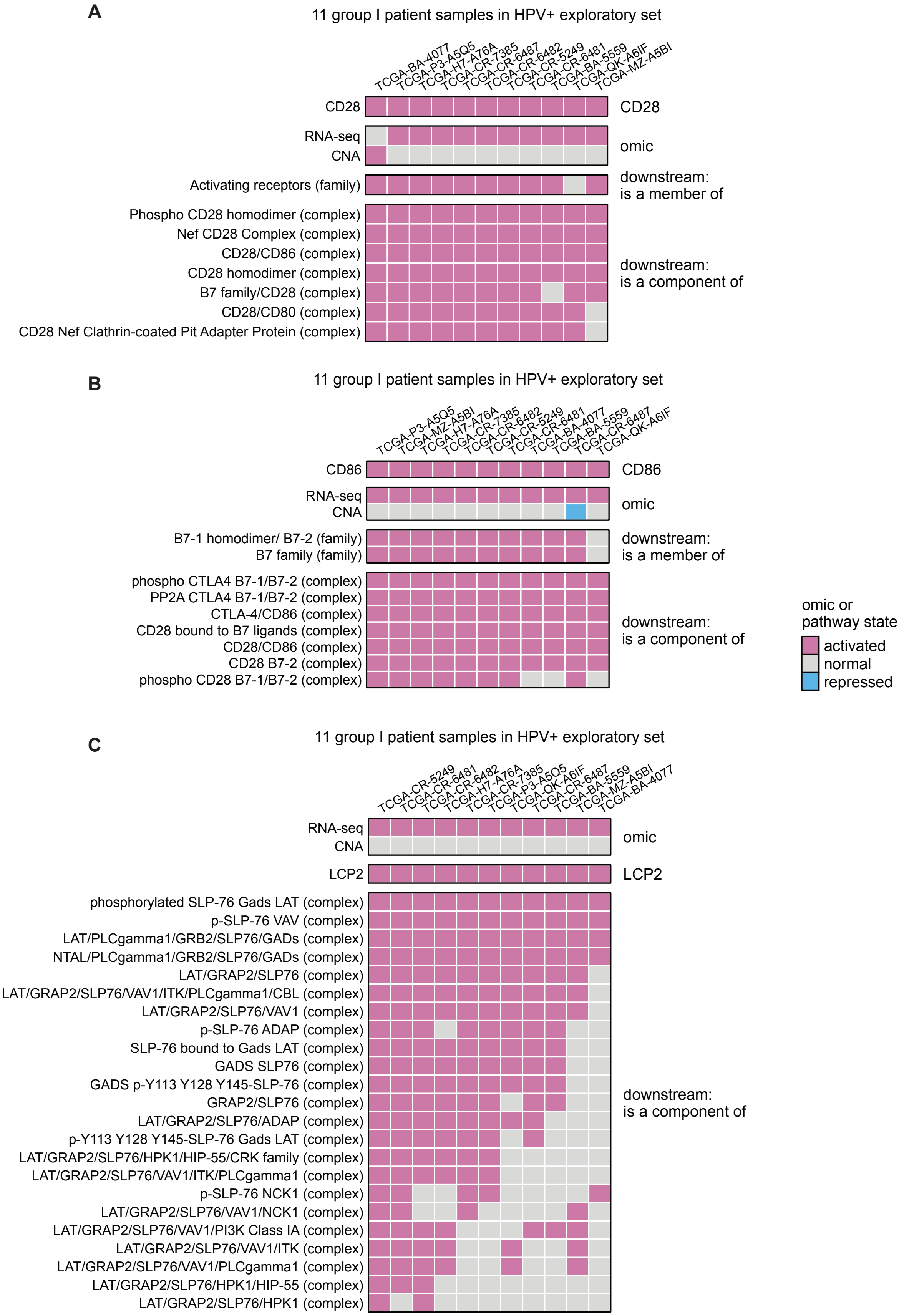

Supplementary Figure 5: CNA, RNA, and pathway states of CD28 (A), CD86 (B), and LCP2 (C), as well as pathway states of their pathway network neighbors in the eleven group I patients.

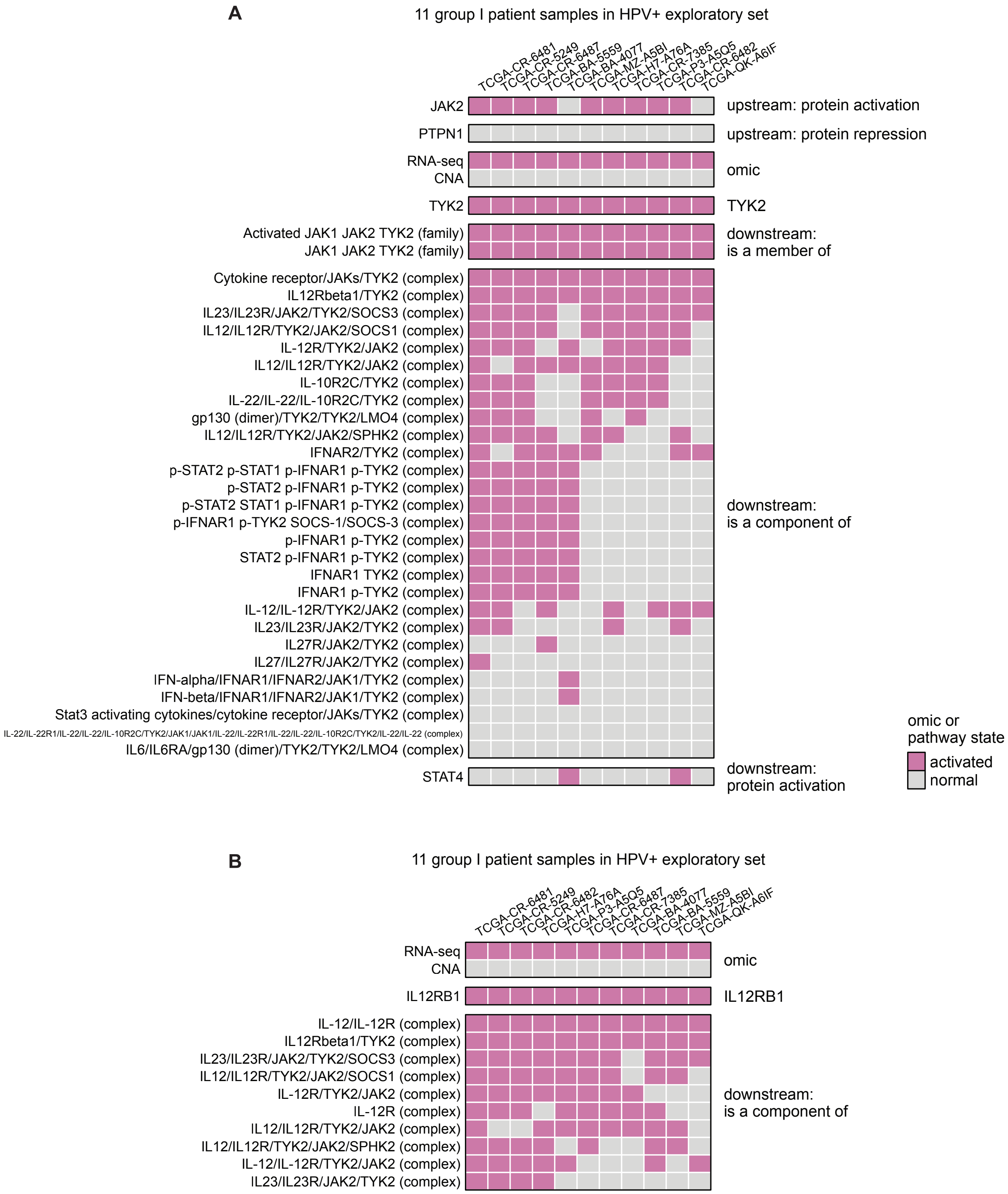

Supplementary Figure 6: CNA, RNA, and pathway states of TYK2 (A) and IL12RB1 (B), as well as pathway states of their pathway network neighbors in the eleven group I patients.

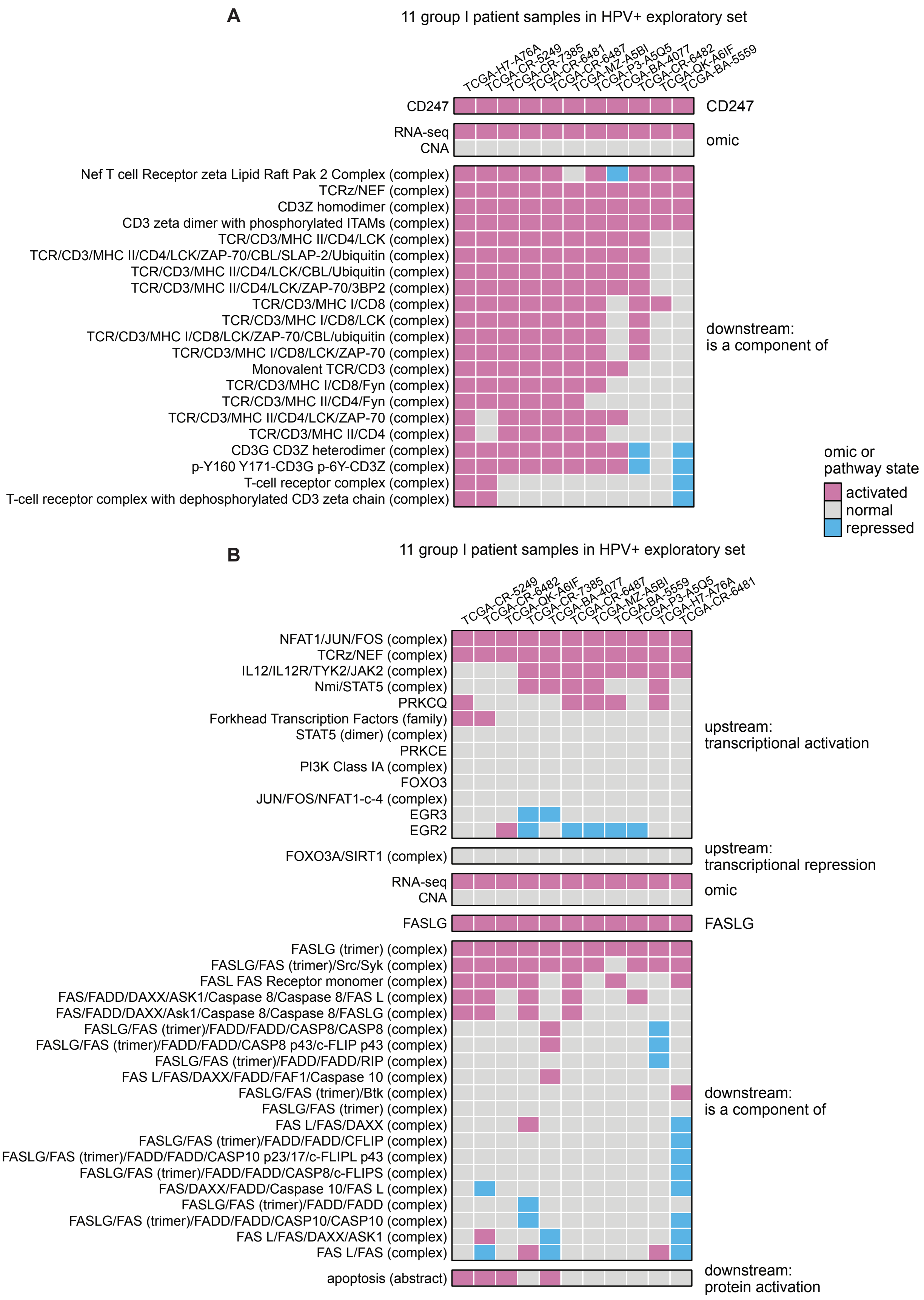

Supplementary Figure 7: CNA, RNA, and pathway states of CD247 (A) and FASLG (B), as well as pathway states of their pathway network neighbors in the eleven group I patients.

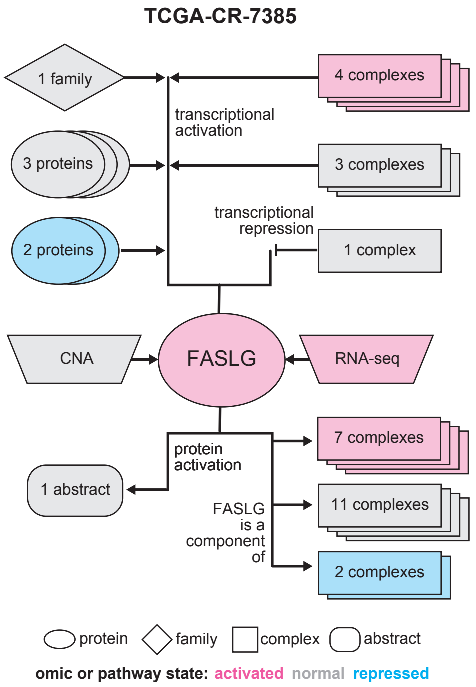

Supplementary Figure 8: CNA, RNA, and pathway states of FASLG as well as pathway states of its pathway network neighbors in a group I patient TCGA-CR-7385.

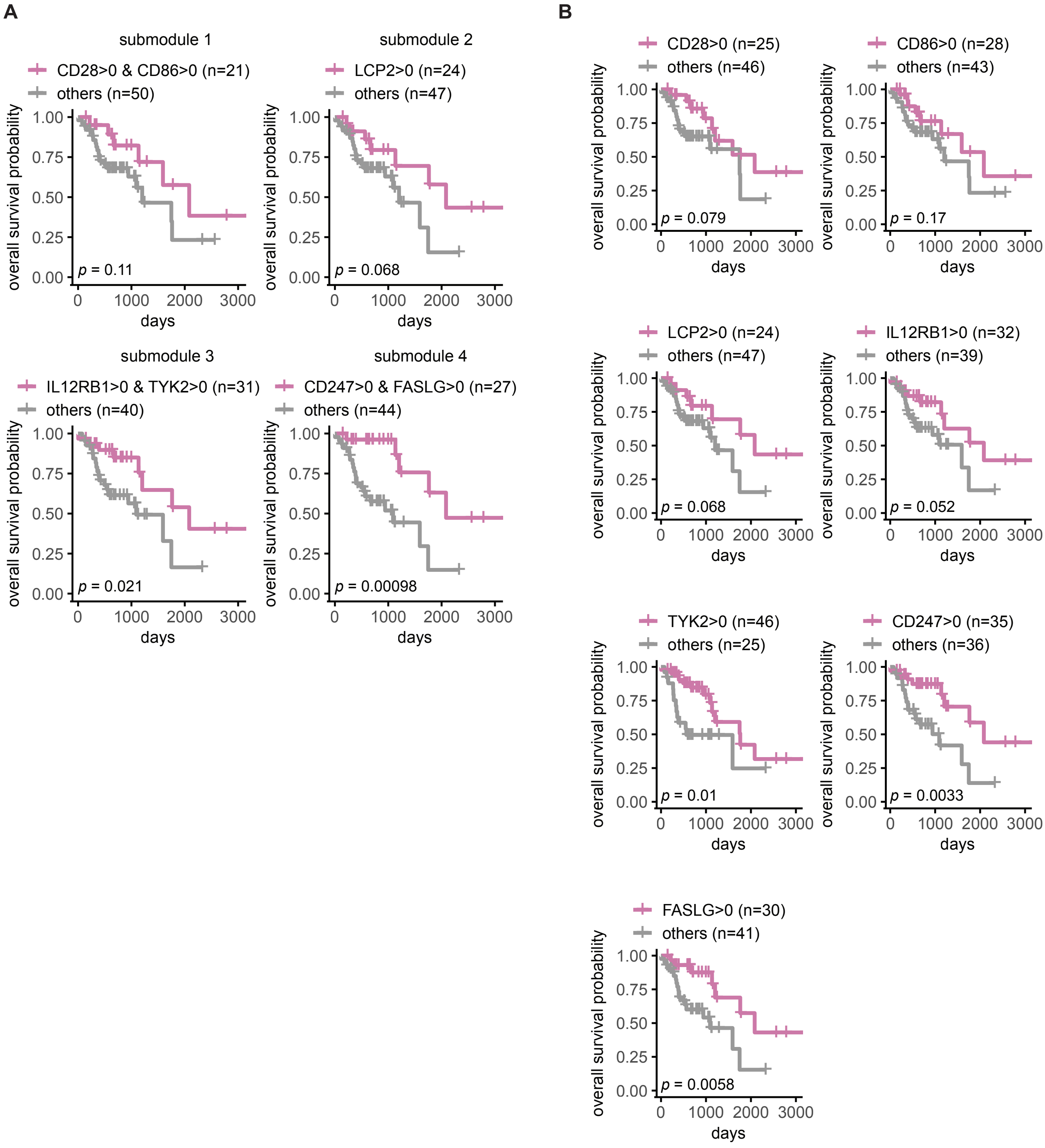

Supplementary Figure 9: Overall survival of HPV+ exploratory set patient samples stratified by the inferred pathway levels (IPLs) of proteins from the same submodule (A) or individual protein (B). Not all overall survival tests were statistically significant under a log-rank $p < 0.01$ cutoff.

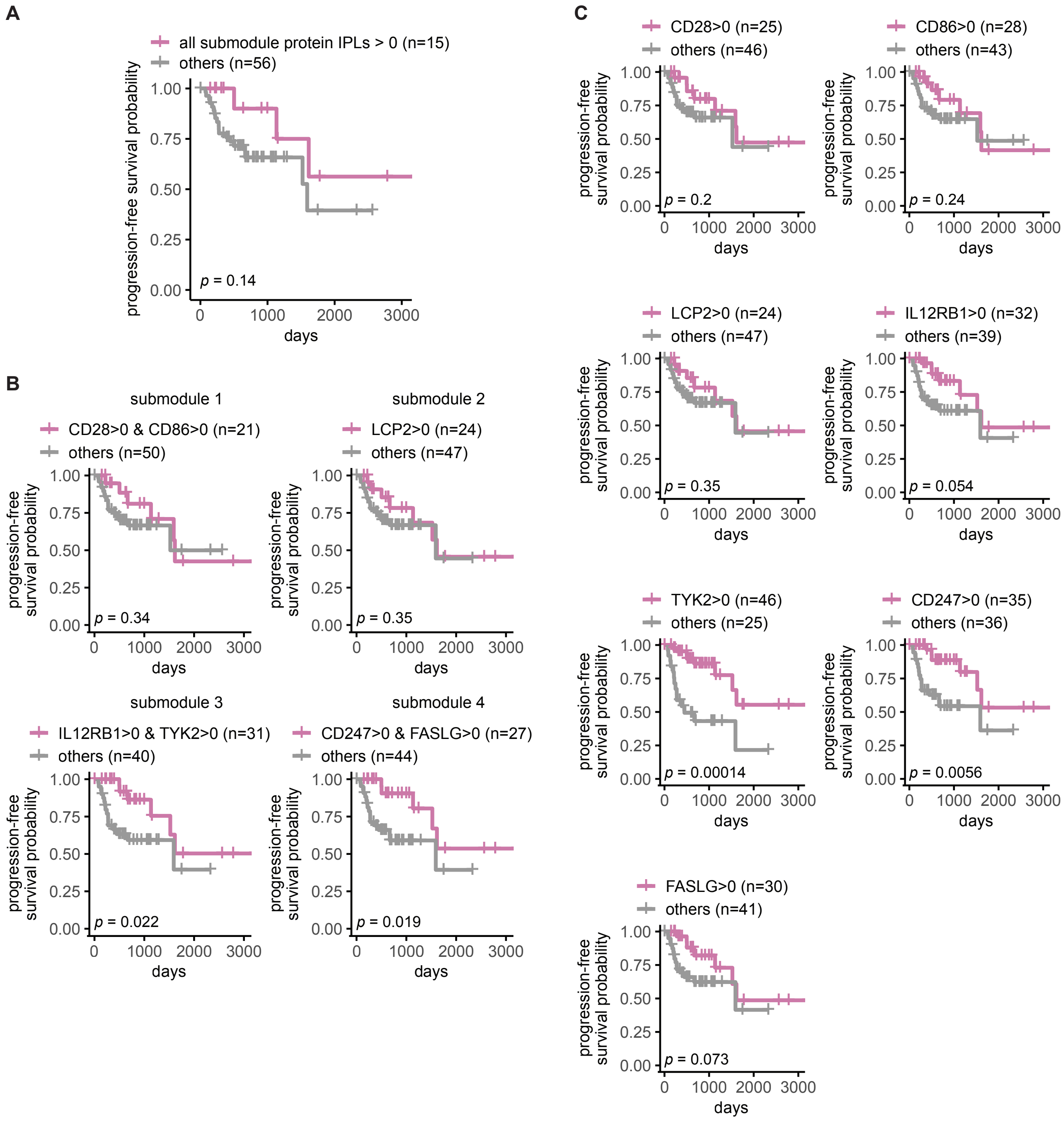

Supplementary Figure 10: Progression-free survival of HPV+ exploratory set patient samples stratified by the inferred pathway levels (IPLs) of all the seven proteins combined (A); proteins from the same submodule (B), or individual protein (C). Not all progression-free survival tests were statistically significant under a log-rank $p < 0.01$ cutoff.

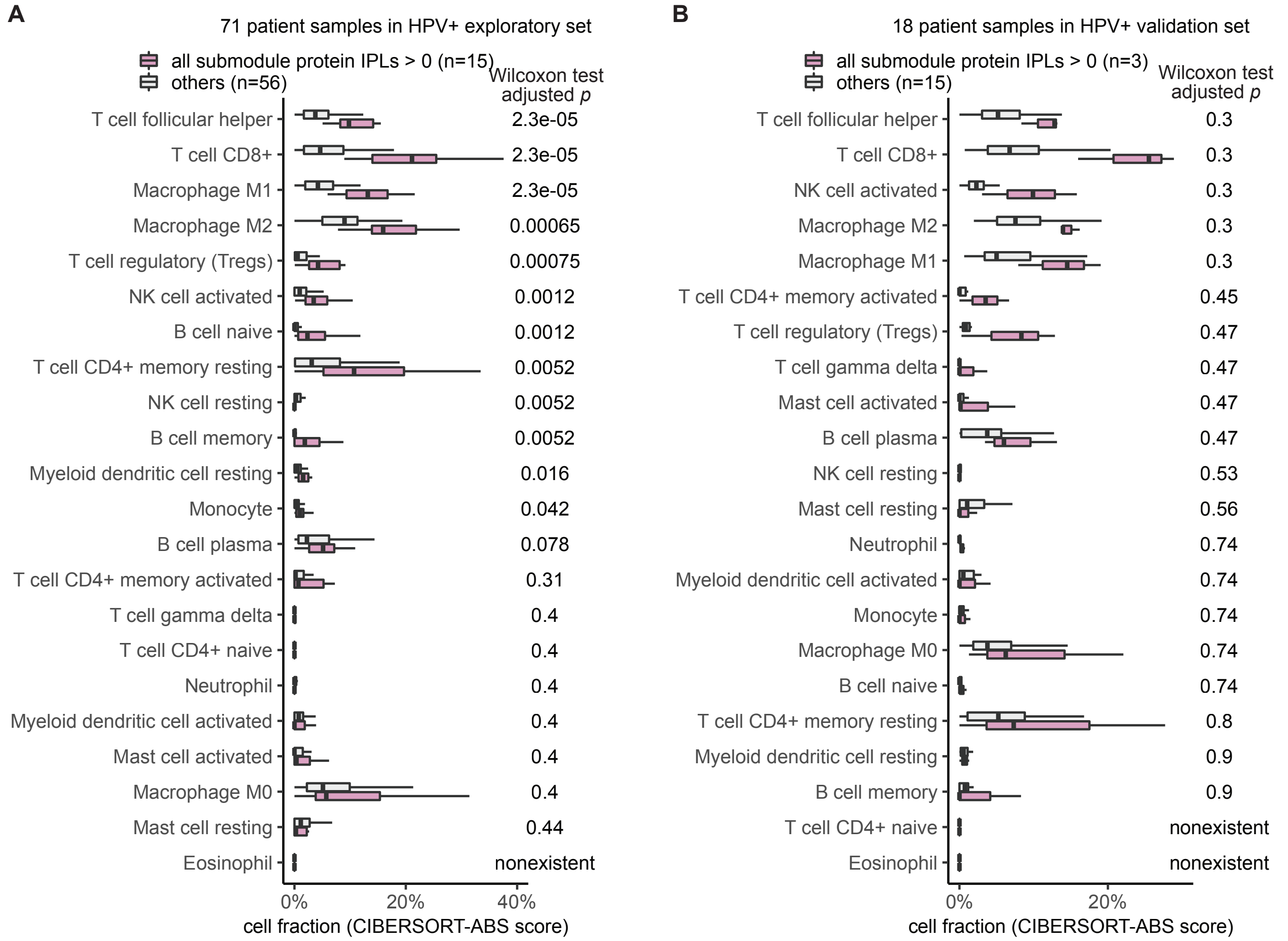

Supplementary Figure 11: Immune cell compositions stratified by the inferred pathway levels (IPLs) of all the seven proteins combined for HPV+ exploratory (A) and validation (B) set. Adjusted Wilcoxon test *p* on the cell composition difference between two groups of patient samples were shown for each cell type. Eosinophil was not found in either the exploratory or validation sets. T cell CD4+ naive was not found in the validation set.

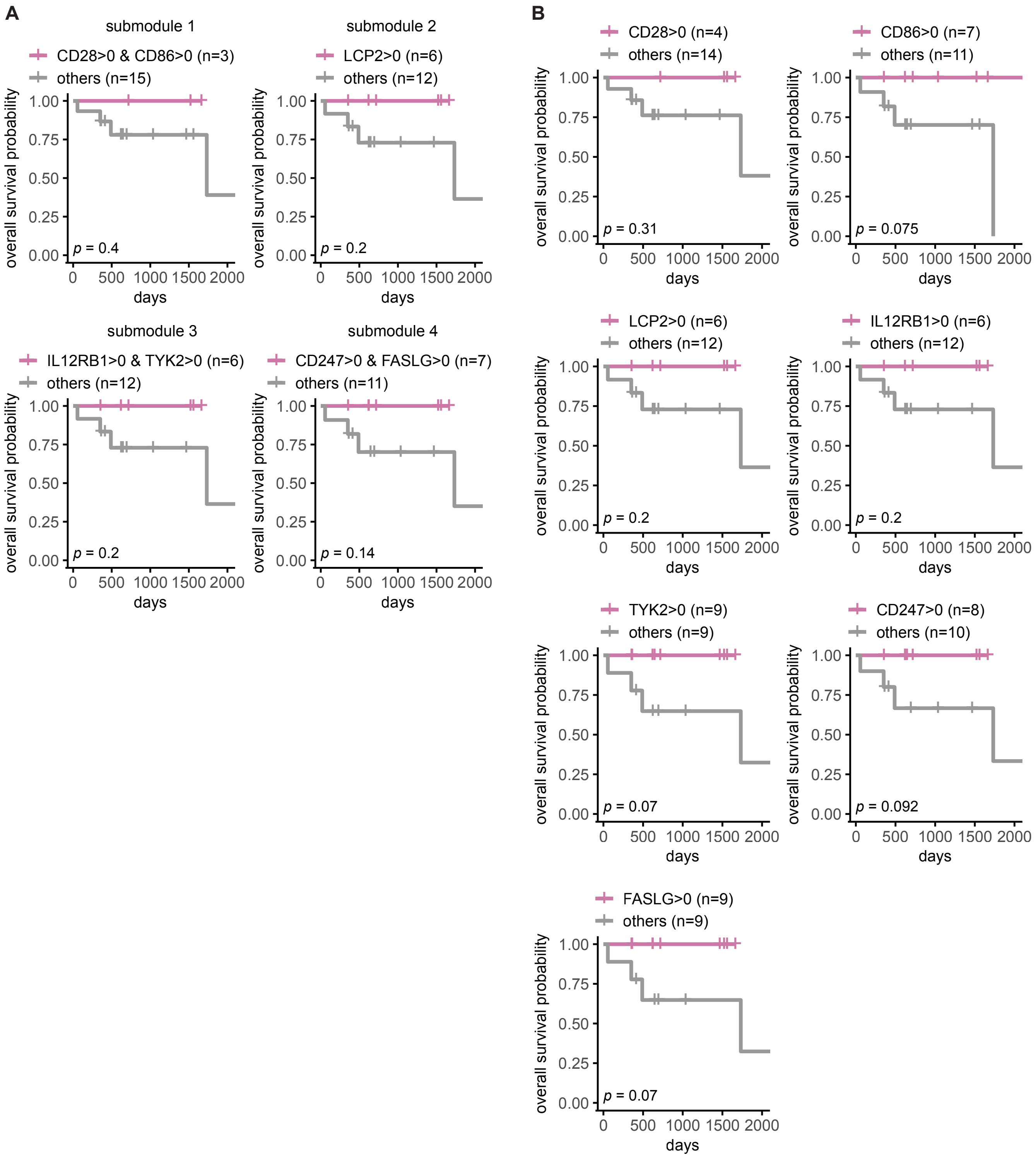

Supplementary Figure 12: Overall survival of HPV+ validation set patient samples stratified by the inferred pathway levels (IPLs) of proteins from the same submodule (A) or individual protein (B). Not all overall survival tests were statistically significant under a log-rank $p < 0.01$ cutoff.

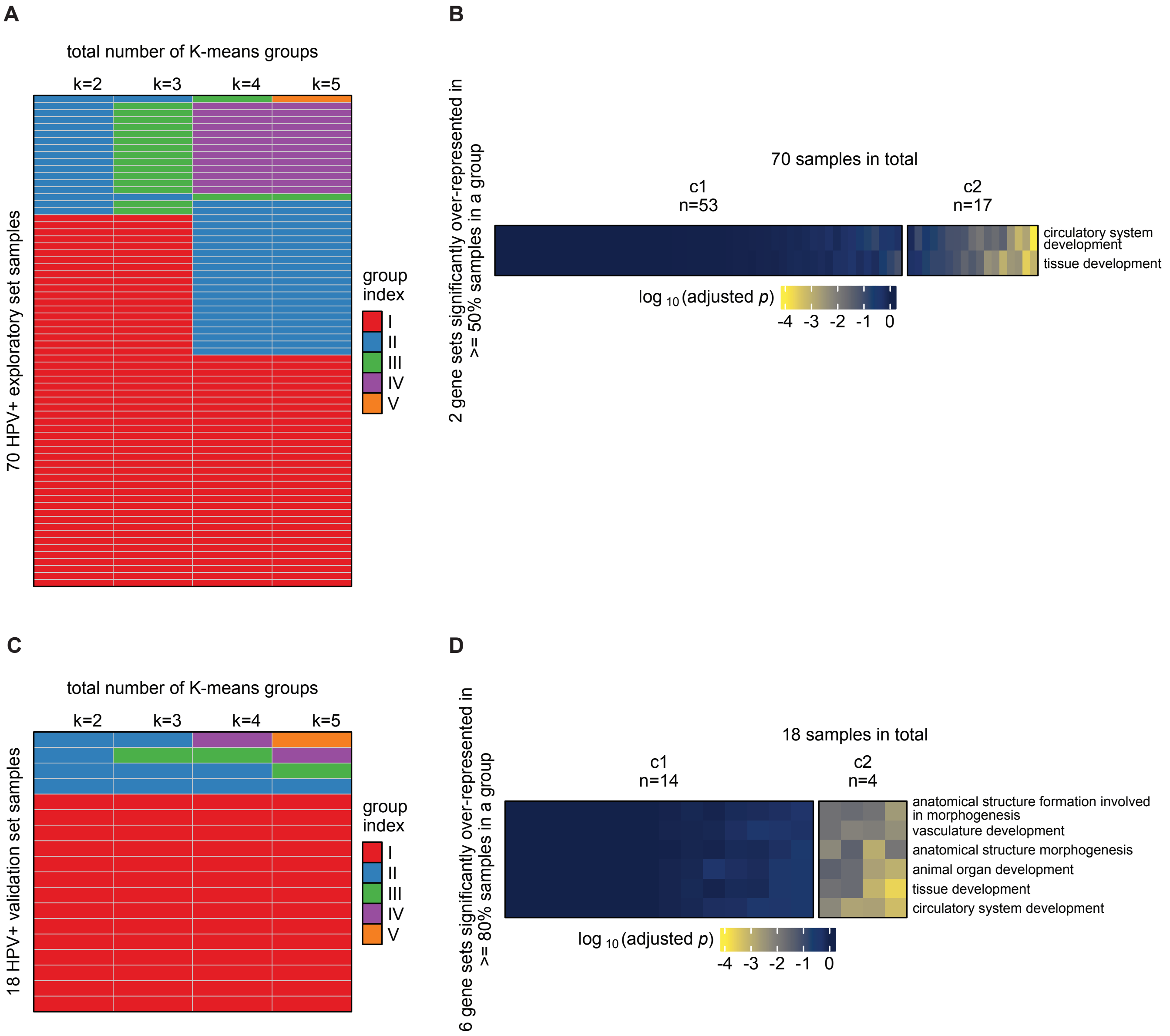

Supplementary Figure 13: Clustering patient samples and annotating patient groups by PARADIGM IPLs from Pan-Cancer Atlas. K-means clustering applied to HPV+ exploratory (A) and validation (C) sets with a total number of groups ranging from two to five. Significantly overrepresented GO terms for groups of patients from HPV+ exploratory (B) and validation (D) sets. Note that one patient sample from HPV+ exploratory set does not have IPLs available in the Pan-Cancer Atlas's PARADIGM results, so only 70 of the 71 samples are shown in A and B.

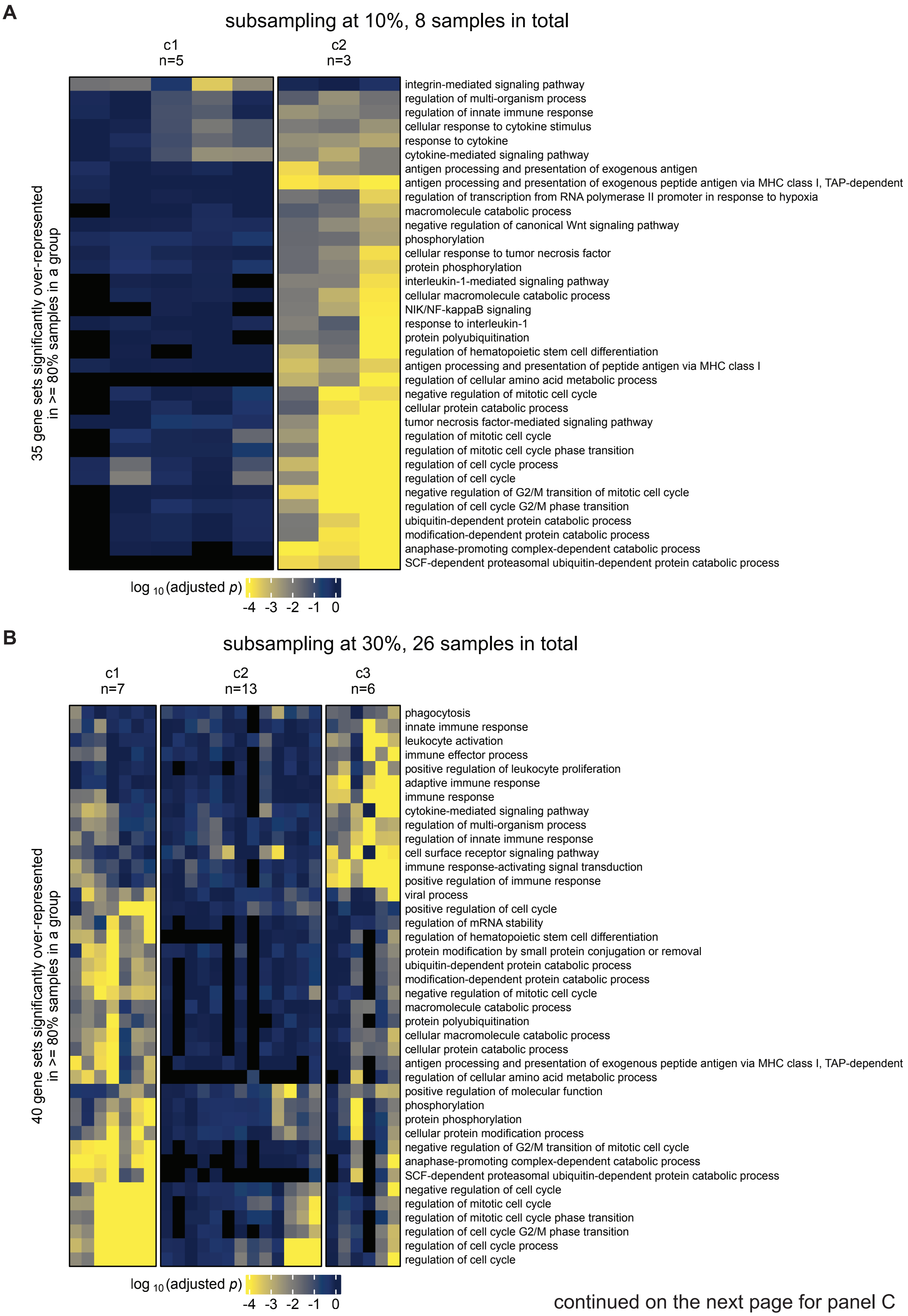

continued on the next page for panel C

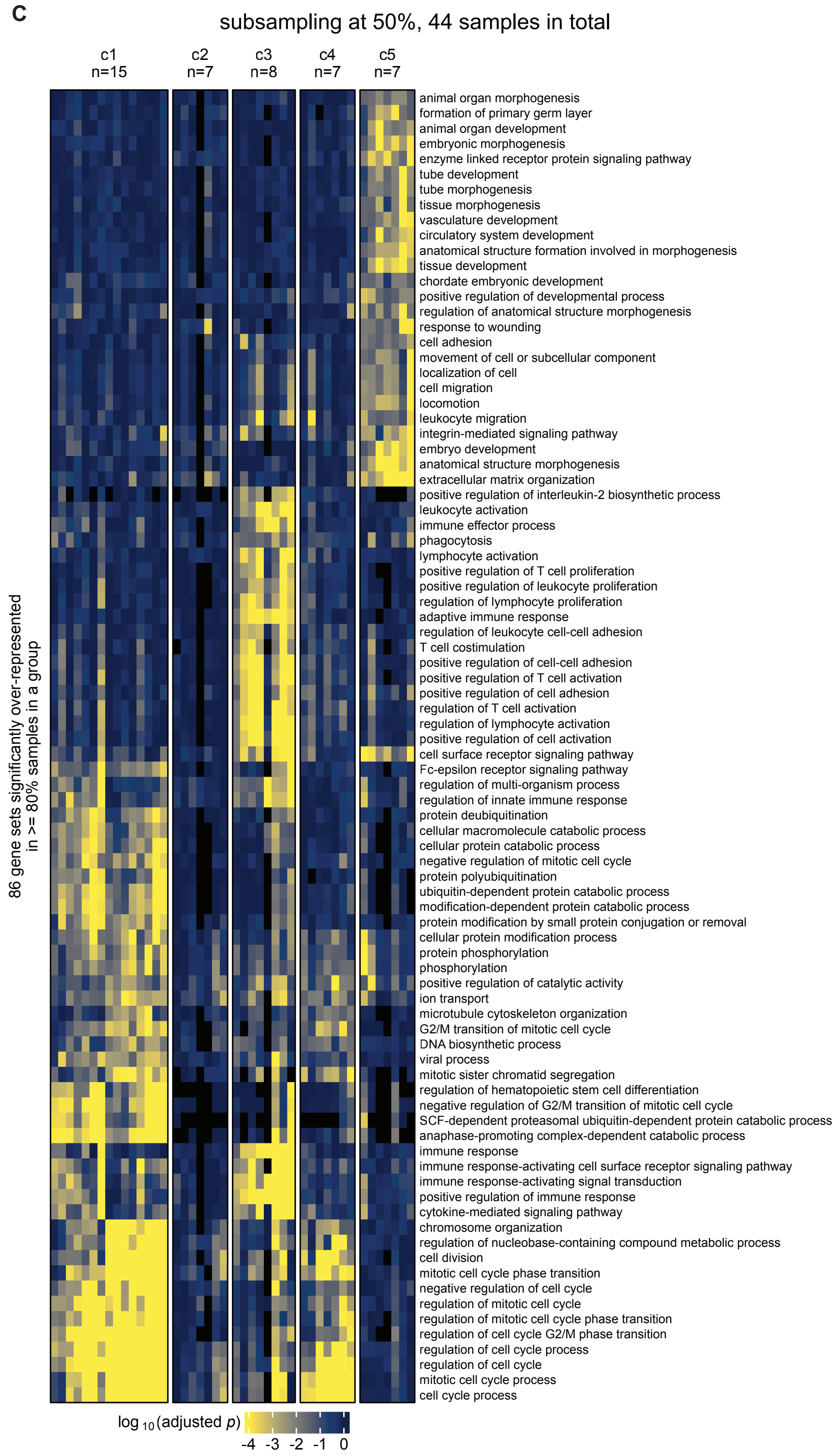

Supplementary Figure 14: Subsampling analysis by MPAC on HPV+ patient samples. Patient groups and their significantly overrepresented GO terms by MPAC on randomly selected 10% (A), 30% (B), and 50% (C) of the 89 samples from HPV+ exploratory and validation set combined.

A

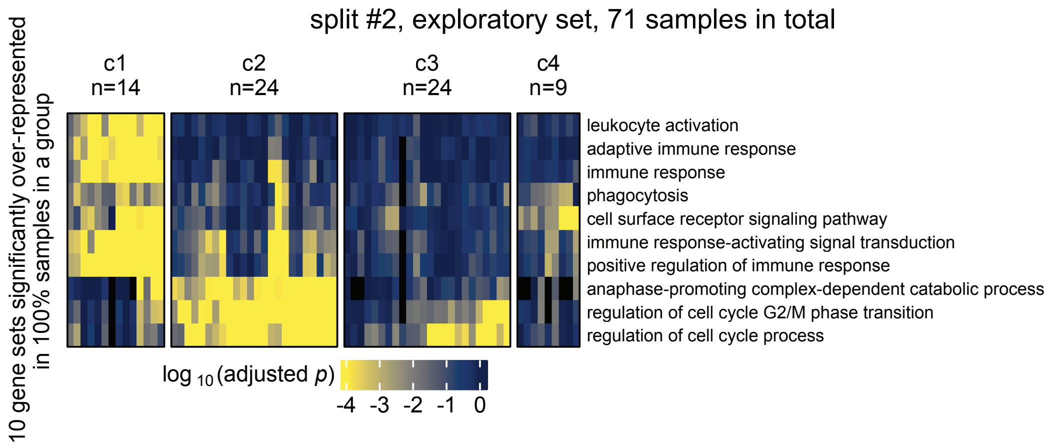

B

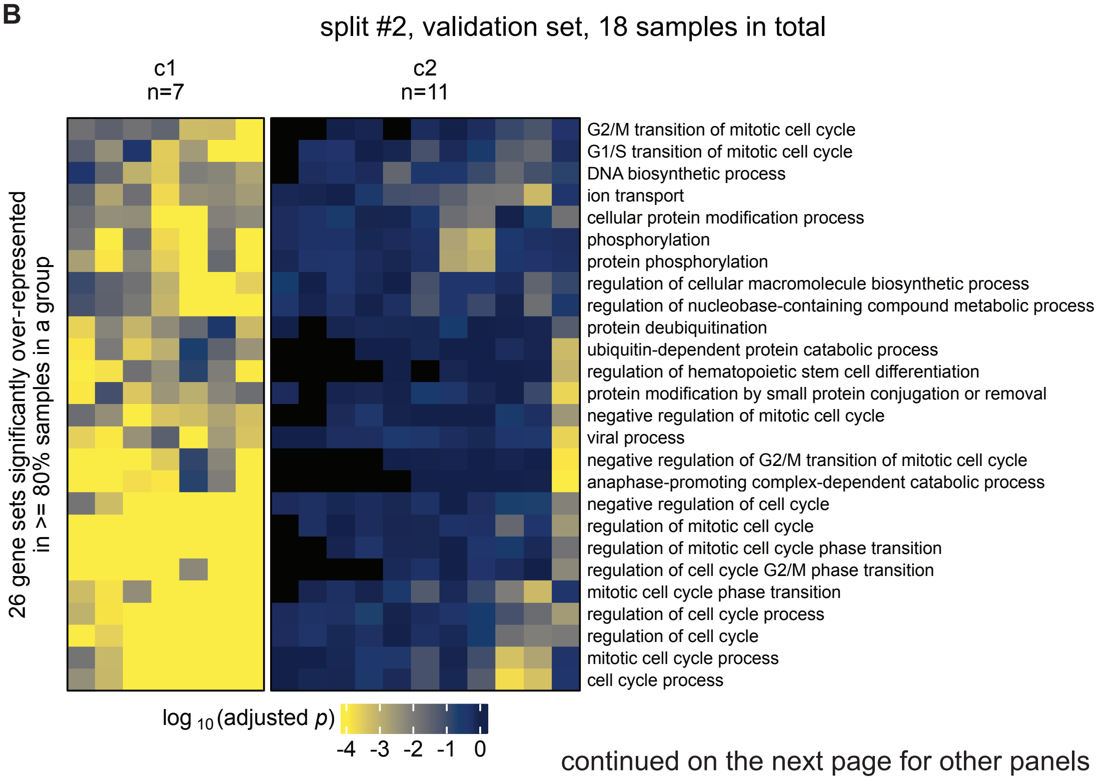

continued on the next page for other panels

C

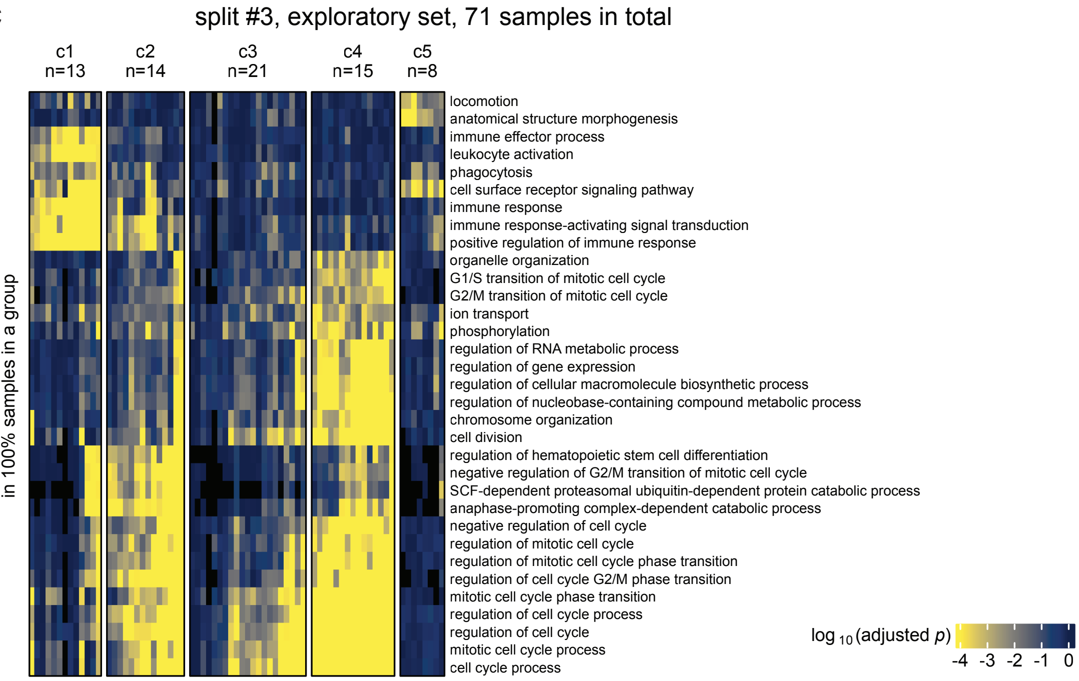

D

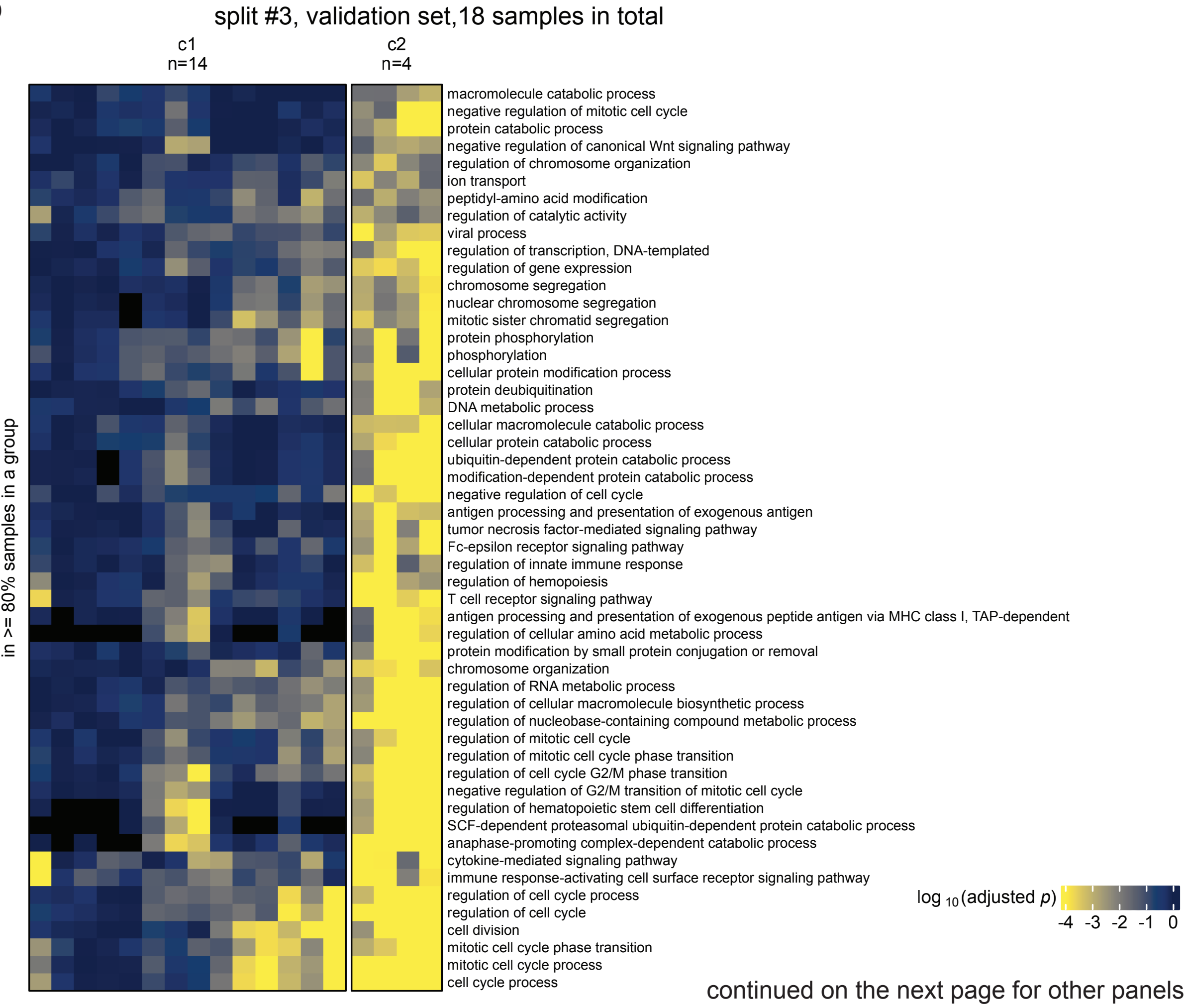

continued on the next page for other panels

E

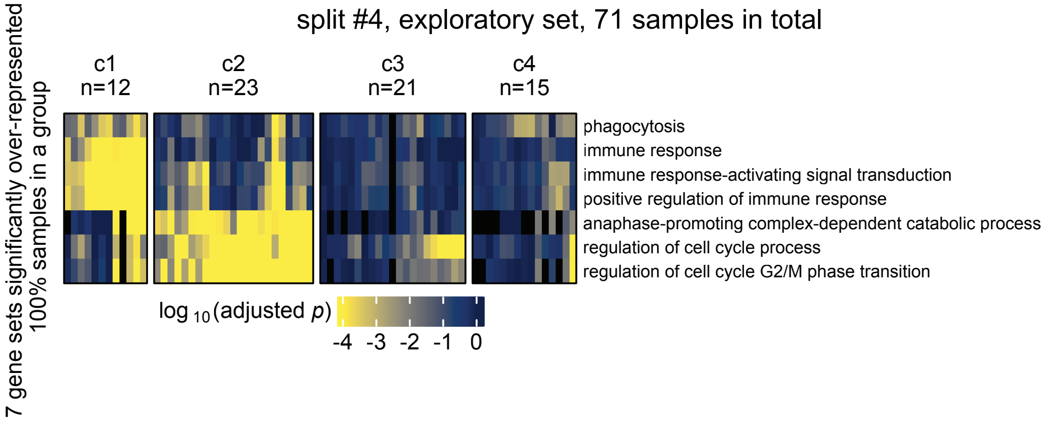

F

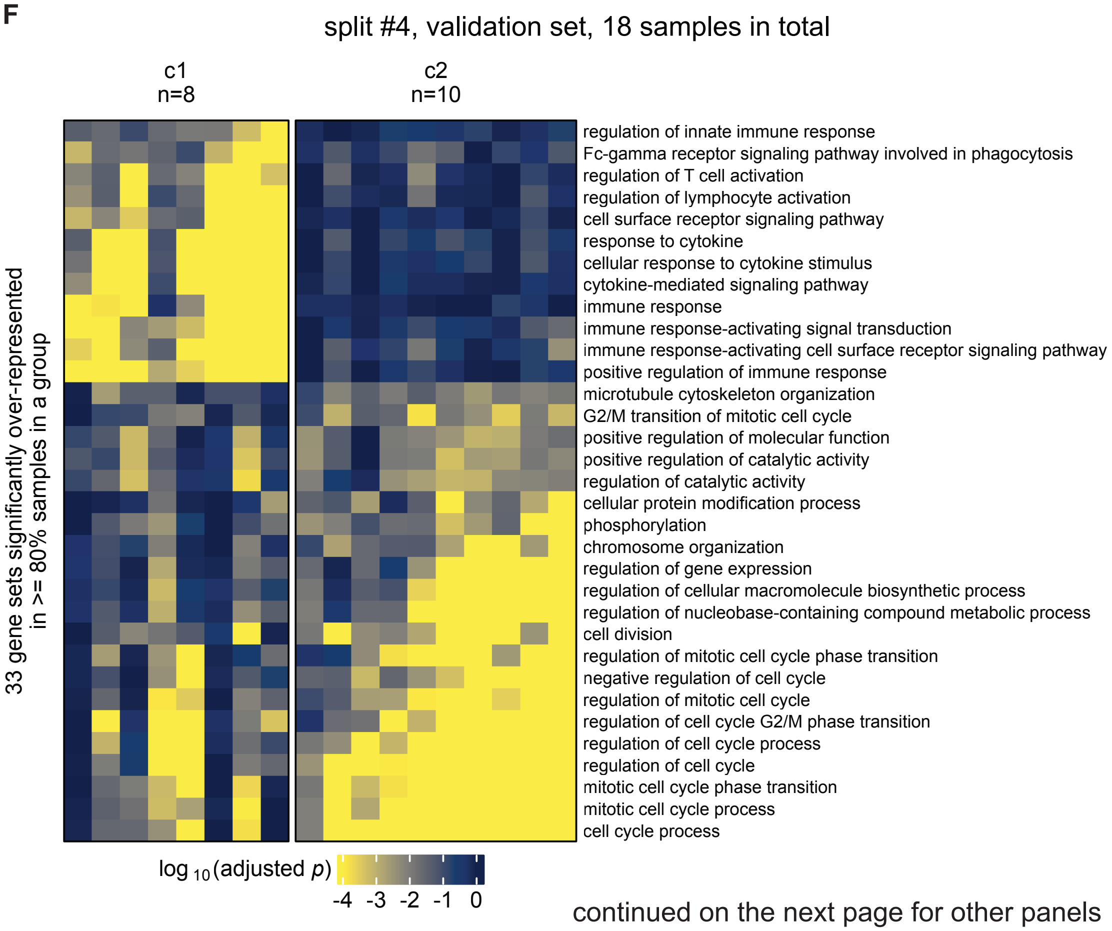

continued on the next page for other panels

G

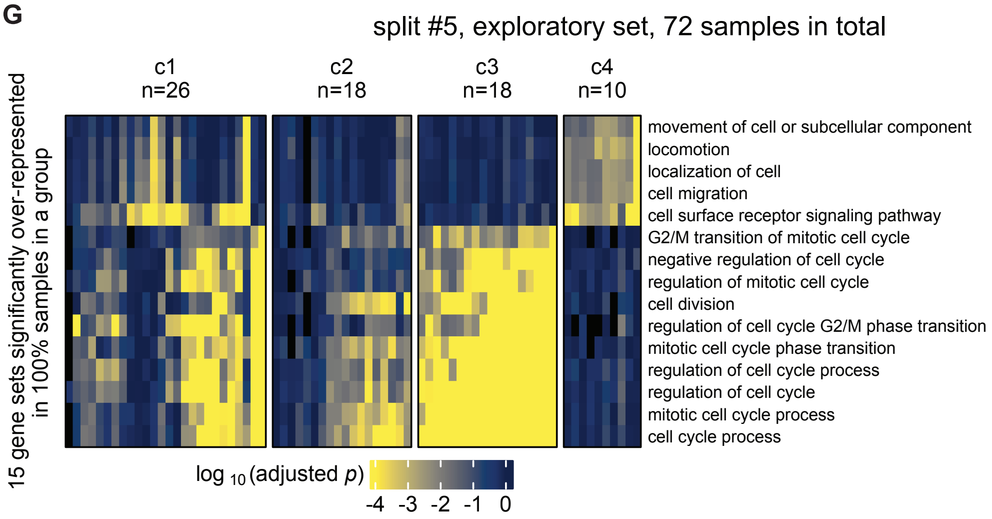

H

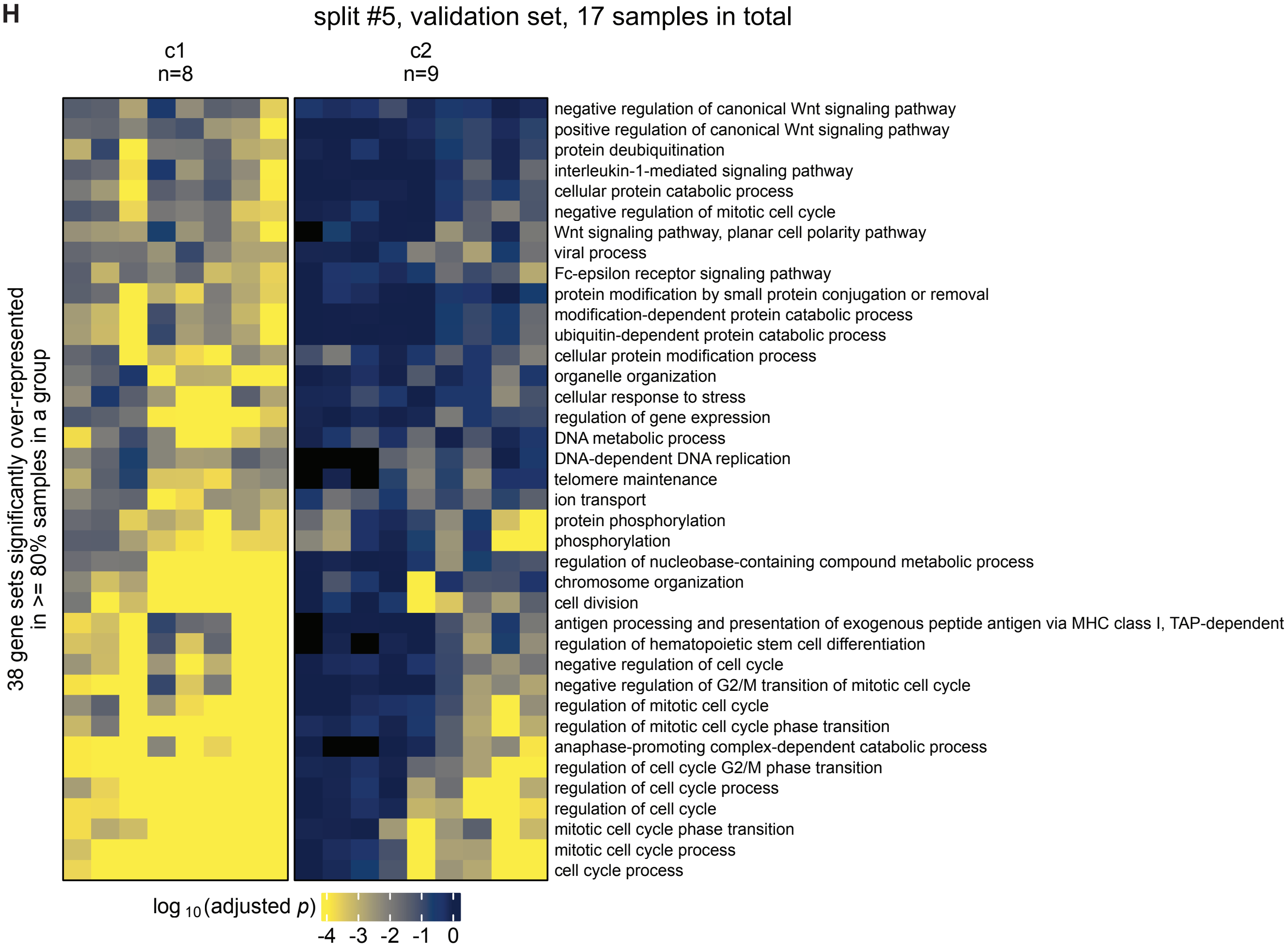

Supplementary Figure 15: Exploratory and validation set resampling of MPAC on HPV+ patient samples. The 71 exploratory set samples were divided into four groups with similar sizes (18, 18, 18, and 17). The 18 validation set samples were used as the fifth group. The existing exploratory set and validation set was defined as split #1. Results for split #1 are shown in Figure 2 and Figure 4A. Patient groups and significantly overrepresented GO terms by MPAC are shown here for split #2 (A & B), #3 (C & D), #4 (E & F), and #5 (G & H).

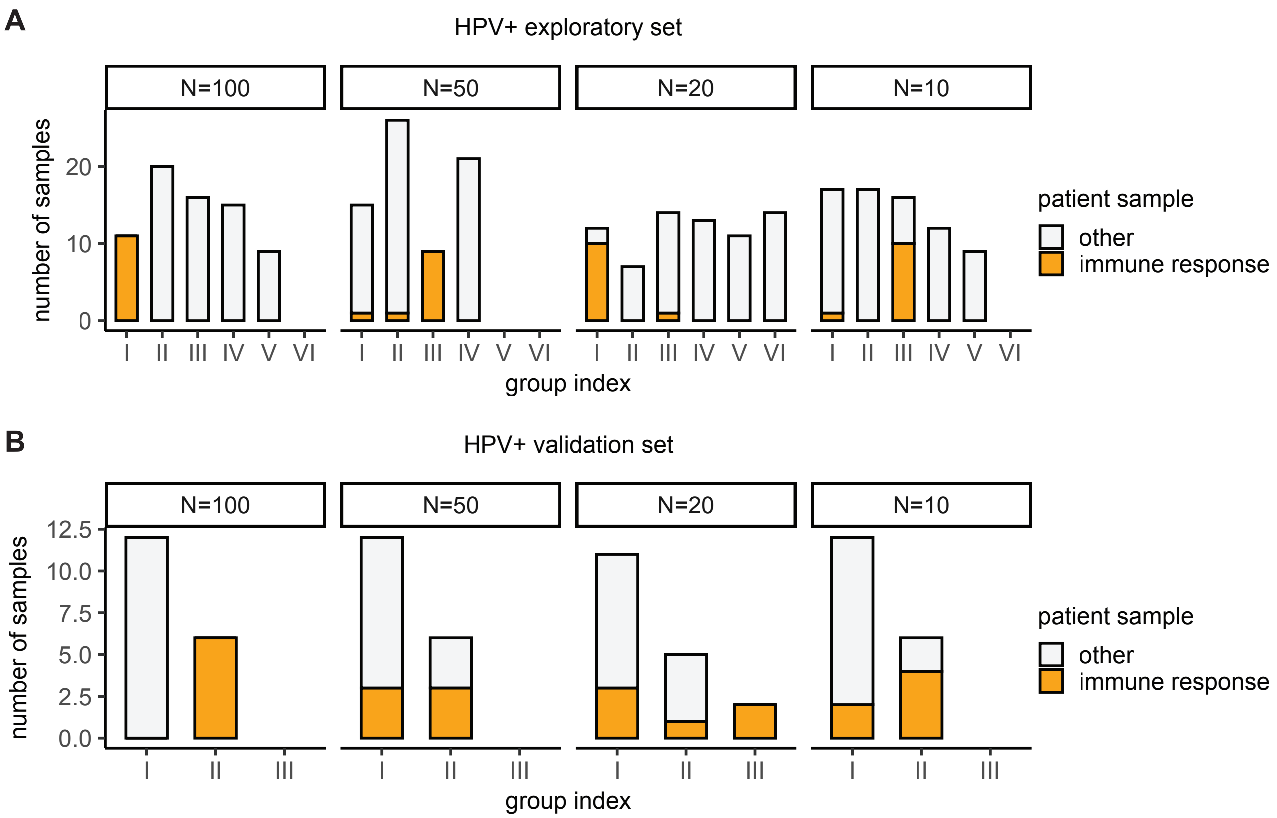

Supplementary Figure 16: Distribution of immune response patient samples under different numbers of permutations for HPV+ exploratory (A) and validation (B) set. Immune response patient samples are defined as the eleven Group I exploratory set samples (Figure 2) and the six Group II validation set samples (Figure 4) from 100 permutations.

A

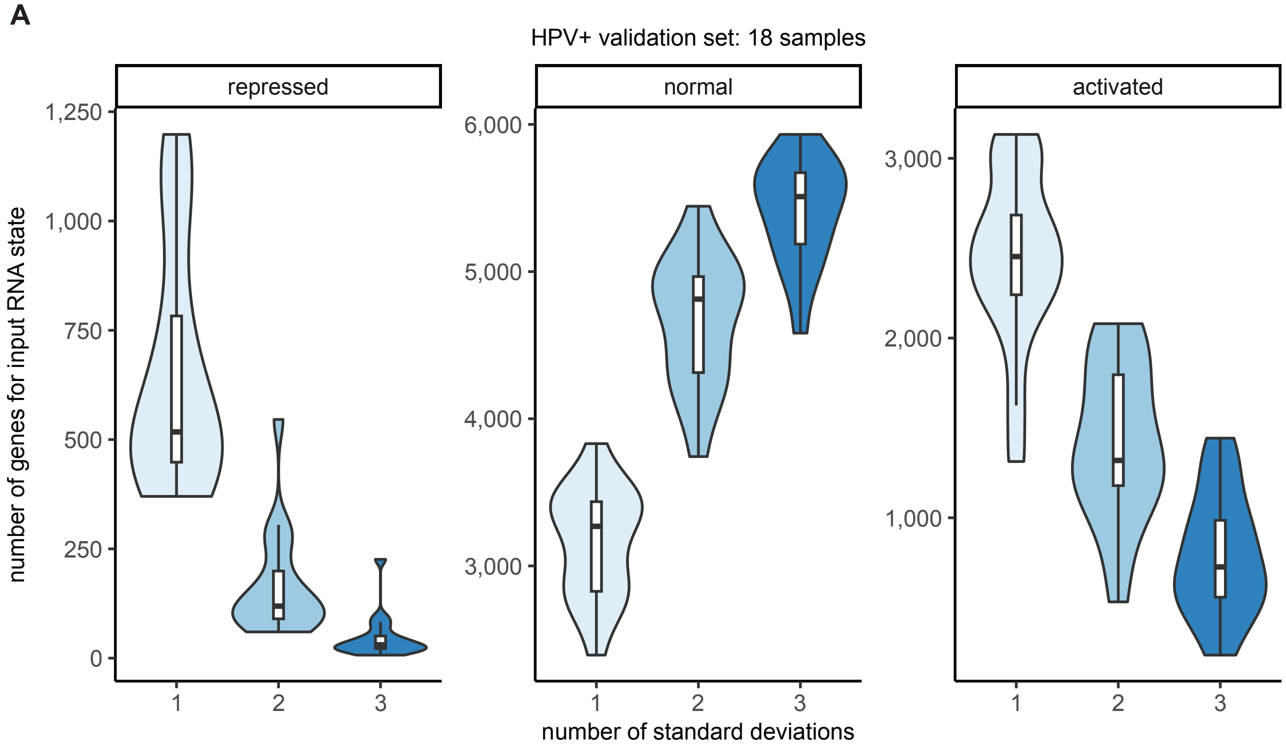

B

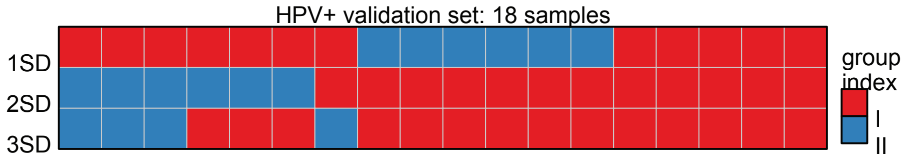

continued on the next page for panel C & D

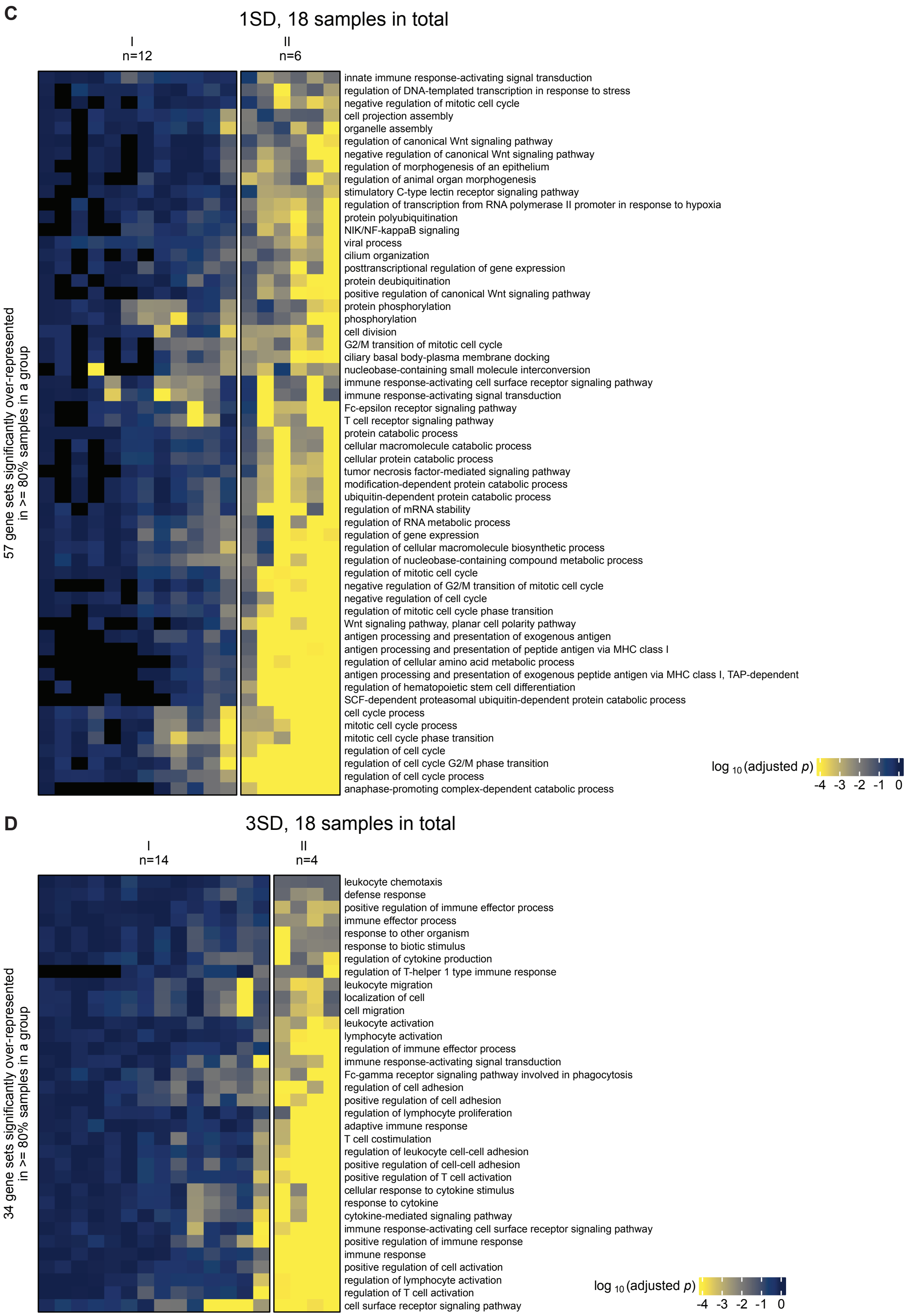

Supplementary Figure 17: Impact of one, two, or three standard deviation thresholds on defining input RNA states for HPV+ validation set. (A) Distributions of input RNA states; (B) Patient groups; (C & D) Significantly overrepresented GO terms in each patient group under one (C) or three (D) standard deviation threshold. Those under two standard deviations are shown in Figure 4A.

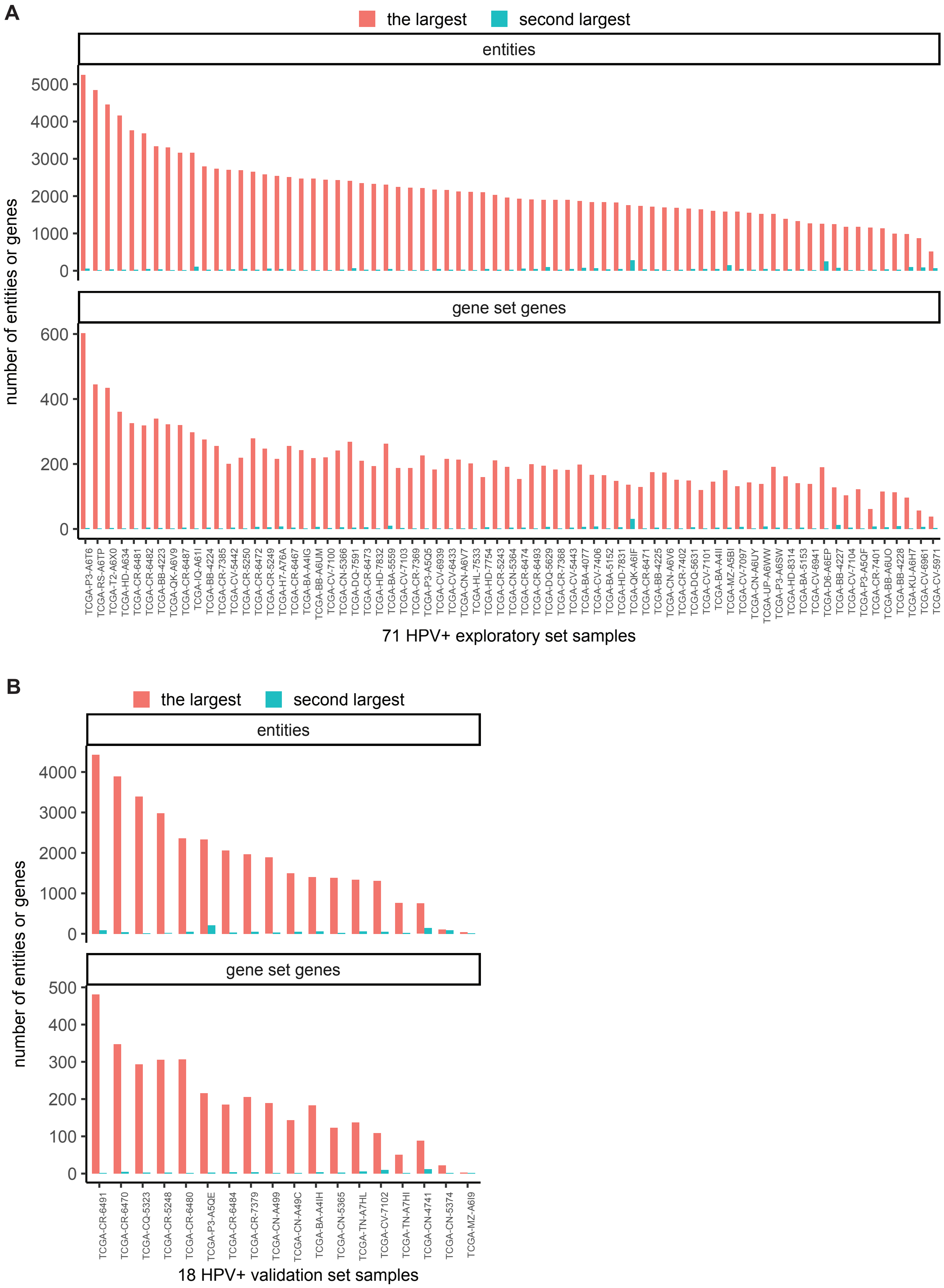

Supplementary Figure 18: Comparison of the sizes of the largest and the second largest sub-network for HPV+ exploratory (A) and validation (B) set. Sizes are represented by the total number of entities or the number of gene set genes that were used for downstream GO term overrepresentation analysis.

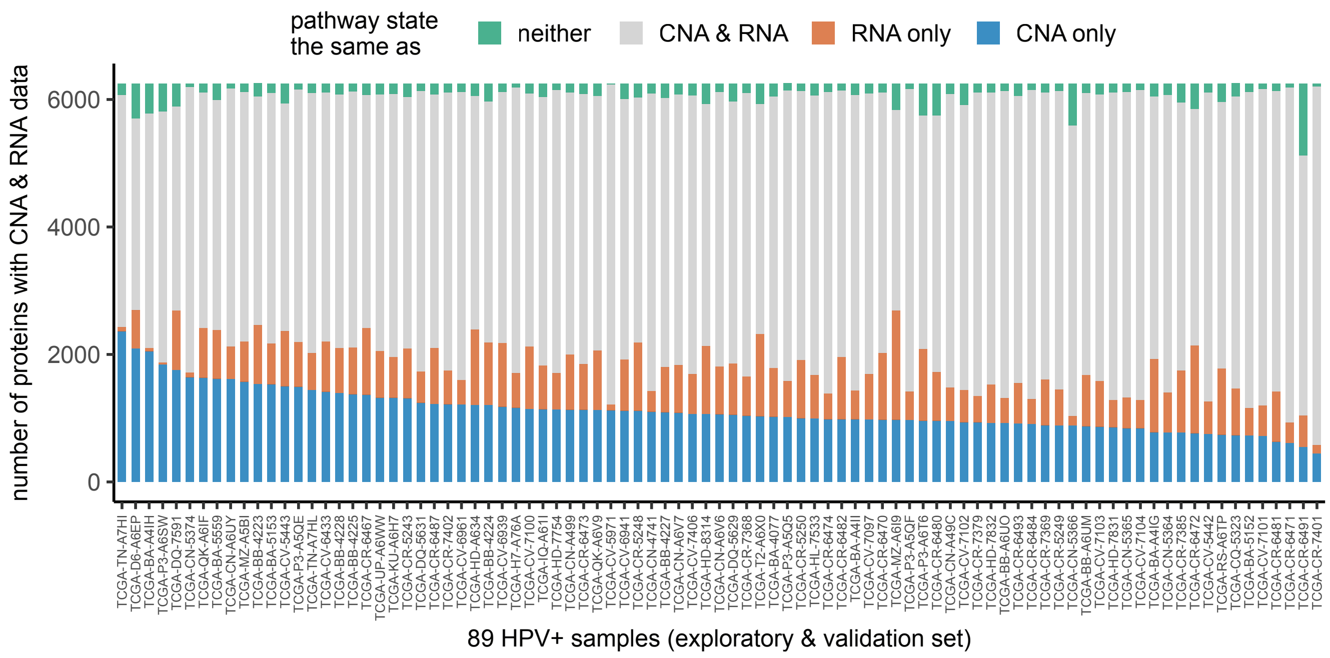

Supplementary Figure 19: Comparison of the number of proteins that have their pathway states agreed with CNA and/or RNA-seq data. All the 6,251 proteins that have CNA and RNA-seq data were stratified by whether their pathway states agree with neither of their CNA or RNA states (green), both CNA and RNA states (gray), only RNA but not CNA states (orange), or only CNA but not RNA states (blue).

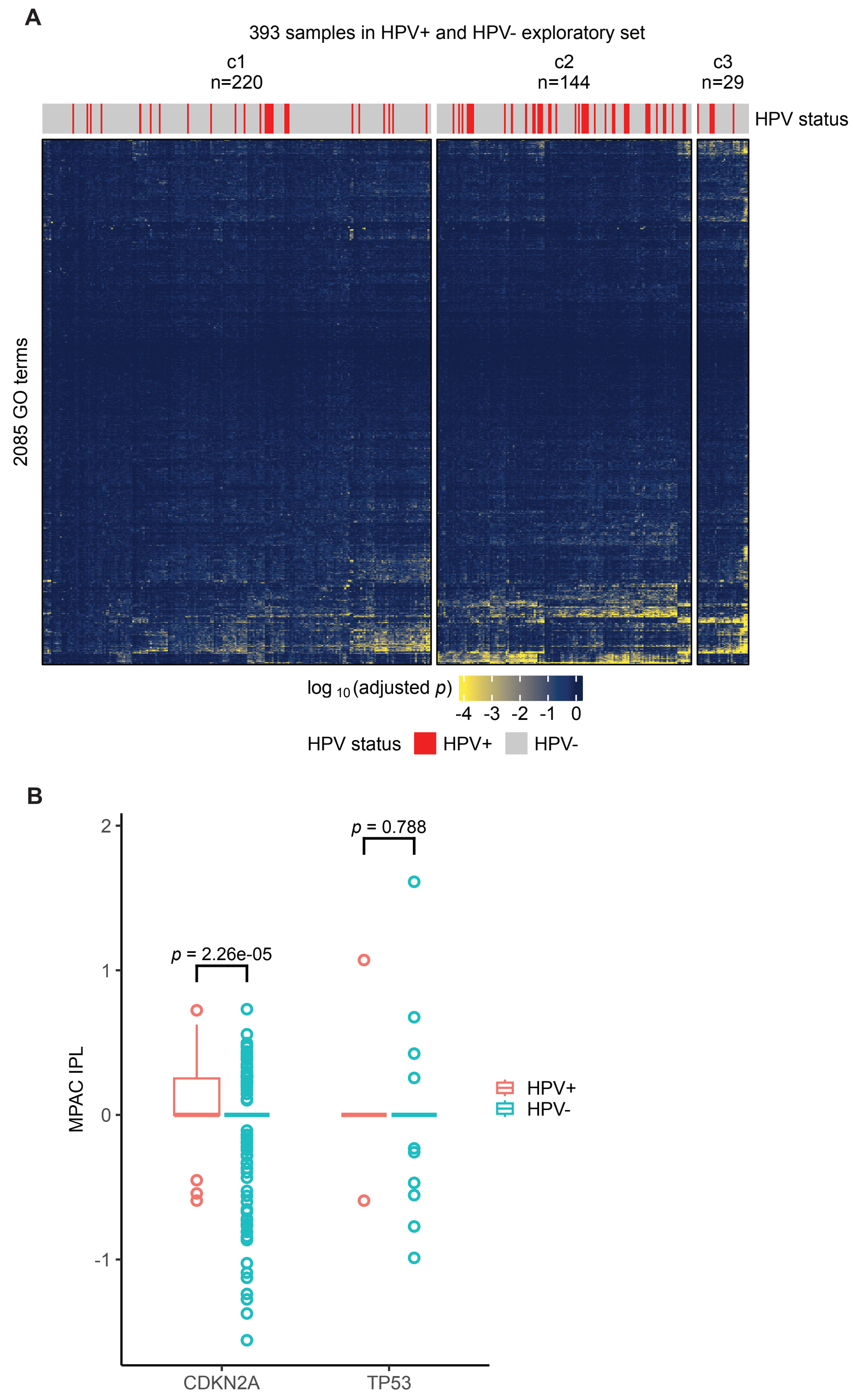

Supplementary Figure 20: MPAC cannot separate HPV+ and HPV- samples due to insufficient input pathway knowledge. (A) MPAC grouping of patient samples from HPV+ (red) and HPV- (gray) exploratory set by their GO term overrepresentations; (B) Comparison of MPAC IPLs of two HPV subtype-specific proteins, CDKN2A and TP53, in patient samples from HPV+ (red) and HPV- (green) exploratory set. Wilcoxon *p*-values on the comparison are denoted.

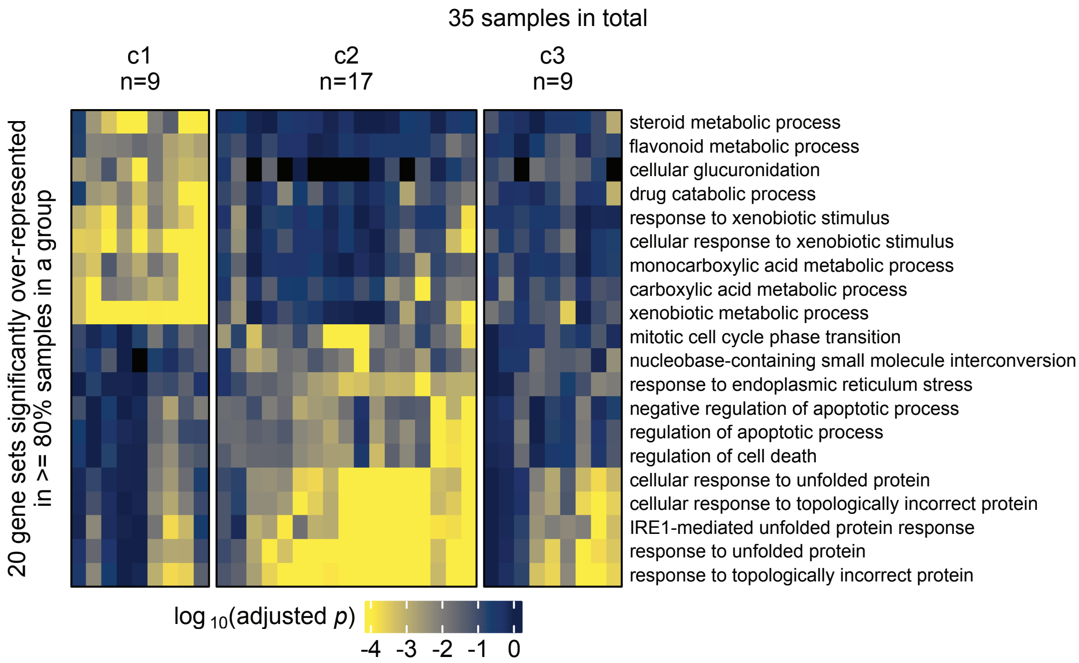

Supplementary Figure 21: Patient sample groups and significantly overrepresented GO terms for the TCGA cholangiocarcinoma cohort.

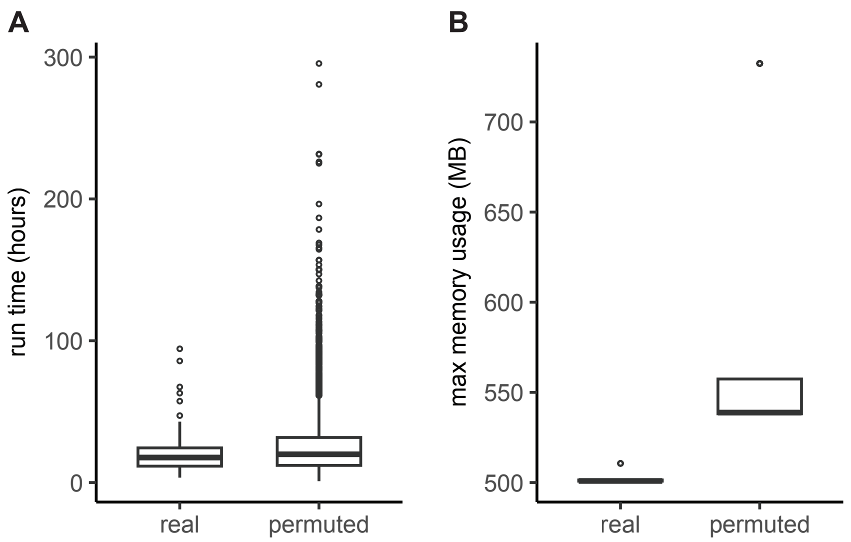

Supplementary Figure 22: Time (A) and memory (B) usage by MPAC's PARADIGM subroutine runs on real and permuted data from the 71 HPV+ exploratory set patient samples. All the jobs were run via HTCondor on machines from the UW–Madison Center for High Throughput Computing.
